# Assessment of Patient-Reported Outcome Measures for Maternal Postpartum Depression Using the Consensus-Based Standards for the Selection of Health Measurement Instruments Guideline: A Systematic Review

**DOI:** 10.1001/jamanetworkopen.2022.14885

**Published:** 2022-06-24

**Authors:** Pervez Sultan, Kazuo Ando, Rania Elkhateb, Ronald B. George, Grace Lim, Brendan Carvalho, Ahish Chitneni, Ray Kawai, Tanya Tulipan, Lindsay Blake, Jessica Coker, James O’Carroll

**Affiliations:** 1 Department of Anesthesiology, Perioperative and Pain Medicine, Stanford University School of Medicine, Stanford, California; 2 Library, University of Arkansas for Medical Sciences, Little Rock; 3 Department of Anesthesiology, University of California, San Francisco; 4 Department of Anesthesiology, University of Pittsburgh, Pittsburgh, Pennsylvania; 5 Physical Medicine and Rehabilitation, NewYork–Presbyterian–Columbia and Cornell, New York, New York; 6 University of California, Davis; 7 Department of Psychiatry, Dalhousie University, Halifax, Nova Scotia, Canada; 8 Department of Psychiatry, University of Arkansas for Medical Sciences, Little Rock

## Abstract

**Importance:**

Maternal depression is frequently reported in the postpartum period, with an estimated prevalence of approximately 15% during the first postpartum year. Despite the high prevalence of postpartum depression, there is no consensus regarding which patient-reported outcome measure (PROM) should be used to screen for this complex, multidimensional construct.

**Objective:**

To evaluate psychometric measurement properties of existing PROMs of maternal postpartum depression using the Consensus-Based Standards for the Selection of Health Measurement Instruments (COSMIN) guideline and identify the best available patient-reported screening measure.

**Evidence Review:**

This systematic review followed the Preferred Reporting Items for Systematic Reviews and Meta-analyses (PRISMA) reporting guideline. PubMed, CINAHL, Embase, and Web of Science were searched on July 1, 2019, for validated PROMs of postpartum depression, and an additional search including a hand search of references from eligible studies was conducted in June 2021. Included studies evaluated 1 or more psychometric measurement properties of the identified PROMs. A risk-of-bias assessment was performed to evaluate methods of each included study. Psychometric measurement properties of each PROM were rated according to COSMIN criteria. A modified Grading of Recommendations Assessment, Development, and Evaluation approach was used to assess the level of evidence supporting each rating, and a recommendation class (A, recommended for use; B, further research required; or C, not recommended) was given based on the overall quality of each included PROM.

**Findings:**

Among 10 264 postpartum recovery studies, 27 PROMs were identified. Ten PROMs (37.0%) met the inclusion criteria and were used in 43 studies (0.4%) involving 22 095 postpartum women. At least 1 psychometric measurement property was assessed for each of the 10 validated PROMs identified. Content validity was sufficient in all PROMs. The Edinburgh Postnatal Depression Scale (EPDS) demonstrated adequate content validity and a moderate level of evidence for sufficient internal consistency (with sufficient structural validity), resulting in a recommendation of class A. The other 9 PROMs evaluated received a recommendation of class B.

**Conclusions and Relevance:**

The findings of this systematic review suggest that the EPDS is the best available patient-reported screening measure of maternal postpartum depression. Future studies should focus on evaluating the cross-cultural validity, reliability, and measurement error of the EPDS to improve understanding of its psychometric properties and utility.

## Key Points

**Question:**

What is the best patient-reported outcome measure (PROM) of postpartum depression based on the Consensus-Based Standards for the Selection of Health Measurement Instruments guideline?

**Findings:**

In this systematic review of maternal postpartum depression screening measures, 10 validated PROMS used in 43 studies involving 22 095 postpartum women were identified. The Edinburgh Postnatal Depression Scale demonstrated adequate content validity and moderate evidence for sufficient internal consistency; the other PROMs demonstrated sufficient content validity but not sufficient internal consistency.

**Meaning:**

The findings suggest that the Edinburgh Postnatal Depression Scale may be the best available PROM of maternal postpartum depression.

## Teaser

This systematic review uses the Consensus-Based Standards for the Selection of Health Measurement Instruments (COSMIN) guideline to evaluate patient-reported outcome measures of postpartum depression and make a recommendation for the best available measure.

## Introduction

Depression is common after childbirth, with the prevalence estimated to be approximately 15% during the first postpartum year.^1,2^ The US maternal mortality rate continues to increase,^3^ and suicide is the second most common cause of maternal mortality in the US.^4,5,6,7^

Early diagnosis and treatment of postpartum depression are desirable to minimize disease severity and associated morbidity. Patient-reported outcome measures (PROMs) are structured questionnaires that allow patients to report their health status. Although PROMs cannot provide a diagnosis of postpartum depression, they are widely regarded as invaluable, inexpensive tools that can be used to screen large numbers of postpartum women in the community setting to identify those at greatest risk of postpartum depression and those who may benefit most from further evaluation and intervention.^8^

The UK National Institute for Health and Care Excellence recommends that clinicians consider using the Edinburgh Postnatal Depression Scale (EPDS) or the Patient Health Questionnaire–9 as part of an assessment of women at risk of developing a mental health problem.^9^ A US Preventive Services Task Force recommendation statement supports risk assessment of women for postpartum depression but provides no recommendations regarding the use of specific PROMs.^10^ There is currently no consensus among national professional societies regarding which PROM should be used to screen for postpartum depression.

A previous scoping review of postpartum recovery measures identified 25 validated PROMs that have been used to evaluate or screen for postpartum depression.^11^ However, the review did not evaluate the methodological quality of the included studies (ie, it did not perform a risk-of-bias assessment) or the overall performance ratings associated with psychometric measurement properties of the PROMs and did not provide levels of evidence supporting the use of PROMs. Systematic reviews are invaluable for summarizing psychometric measurement properties (measures of validity, reliability, responsiveness, and feasibility) of PROMs used to evaluate different postpartum recovery domains and for providing evidence-based recommendations regarding which PROM to use in clinical settings and in future postpartum studies.^12,13,14,15^ Identifying optimal PROMs may also help to inform future study design, highlight knowledge gaps in the literature relating to psychometric measurement properties of existing PROMs, and assess the need to develop new measures. In this systematic review, we used the Consensus-Based Standards for the Selection of Health Measurement Instruments (COSMIN) guideline^16^ to evaluate the performance ratings of psychometric measurement properties of validated PROMs used to screen for maternal postpartum depression based on levels of evidence supporting their use and aimed to make recommendations regarding the best available measure.

## Methods

This systematic review of PROMs used to evaluate postpartum depression followed the Preferred Reporting Items for Systematic Reviews and Meta-analyses (PRISMA) reporting guideline^17^ and COSMIN guideline^16^ and was prospectively registered with PROSPERO (CRD42020178620). Because domains of postpartum depression have not been proposed for evaluating content validity, postpartum recovery and depression experts among us (P.S., R.B.G., G.L., B.C., T.T., J.C., and J.O.) developed a list of domains that were deemed most relevant to maternal postpartum depression. The proposed list of domains was developed based on literature review, professional society criteria (*Diagnostic and Statistical Manual of Mental Disorders* [Fifth Edition] [*DSM-5*]),^18^ and clinical reasoning. Experts among us (P.S., R.B.G., G.L., B.C., T.T., J.C., and J.O.) agreed on the following 4 domains of postpartum depression: affective, behavioral, somatic, and interference. eTable 1 in the Supplement summarizes the proposed domains of postpartum depression and the individual symptoms classified in each domain.

### Search Strategy

A medical librarian (L.B.) searched the literature using the following databases: PubMed, CINAHL, Embase, and Web of Science. The PROMs used to evaluate postpartum depression were identified in a previous scoping review of measures used to evaluate recovery after all modes of delivery.^11^ The PROMs were grouped into different postpartum recovery domains, including psychosocial distress (postpartum depression, anxiety, and other psychological morbidity), as previously described.^12,19^ The comprehensive search strategy from the previous scoping review^11^ was performed with no date limiters on July 1, 2019 (eAppendix 1 in the Supplement), and a second search including a hand search of references from eligible studies was conducted in June 2021. The studies identified in the previous scoping review^11^ that used validated PROMs of postpartum depression were subsequently retrieved and screened in detail to identify the PROMs and studies that met our inclusion criteria. The initial search identified 27 PROMs used to evaluate postpartum depression.

### Inclusion and Exclusion Criteria

The PROMs reported in the previous scoping review^11^ were evaluated in detail to identify those that (1) consisted of at least 2 questions and (2) had undergone some form of psychometric property evaluation (validation study) in a population of women after childbirth. Self-reported measures using isolated numerical values chosen by patients to report depression severity (using a verbal reporting or visual analog scale [eg, 0-10 or 0-100]) were excluded because these measures involve only 1 question or item.

We included any validation study that assessed 1 or more psychometric measurement properties of a given PROM of postpartum depression. We included all types of study design that assessed any of the following 8 psychometric measurement properties defined in the COSMIN guideline in the postpartum population^16^: (1) structural validity (whether the score from a PROM reflected the dimensions of postpartum depression); (2) internal consistency (how strongly the individual items were related in a given PROM); (3) cross-cultural validity (how items of a PROM performed in a translated or culturally adapted version compared with the original version) and/or measurement invariance (similarity of item responses by women from different patient groups); (4) reliability (whether PROM scores remained unchanged for patients whose clinical condition remained unchanged); (5) measurement error (in which systemic and random errors of individual scores were not attributed to true changes in postpartum depression); (6) criterion validity (the degree to which a PROM score was an adequate reflection of the gold standard measure of postpartum depression, the Structured Clinical Interview for *DSM-5* [SCID-5], or of clinical diagnosis by an appropriately trained professional); (7) hypothesis testing (the degree to which the scores of a PROM were consistent with a hypothesis [eg, difference in scores among women with a preexisting mental health disorder]); and (8) responsiveness (the ability of a PROM to detect change at different time points).

We excluded studies that used the PROM as an isolated outcome measure and studies that failed to assess any of the 8 psychometric measurement properties. We excluded studies in which the PROM was designed to be clinician led or in which the instruments were not based on symptoms. We also excluded studies that evaluated only antenatal depression or paternal depression, failed to use or report the use of PROMs in their entirety (eg, studies that used only a portion or unvalidated short form of a PROM), were not published in English, and were published as abstracts, letters, theses, or editorials.

### Data Extraction

Duplicates from different databases were removed, and the remaining articles were entered into the Rayyan online reviewing system for evaluation.^20^ Four of us (P.S., K.A., R.K., and J.O.) determined whether studies met the inclusion criteria for this review. Conflicts were resolved after discussion with a fifth one of us (B.C.). A standardized database was used to extract data from the included studies.

### Data Analysis

We analyzed data from identified validation studies as recommended in the COSMIN guideline.^16^ Analysis involved PROM assessment in 7 steps.^16^ First, content validity was assessed by reporting the number of proposed postpartum depression domains (eAppendix 2 in the Supplement) evaluated by each PROM. A PROM was considered to have adequate content validity if it evaluated at least 3 of the 4 proposed domains relevant to postpartum depression (ie, it assessed ≥75% of the proposed domains). Second, feasibility for the use of each PROM was assessed by evaluating ease of accessibility, cost of use for research purposes, response rates from individual studies, completeness of returned PROMs (missingness), and documented time taken to complete the PROM.

Third, the methodological quality (risk of bias) of the studies’ assessment of each psychometric measurement property was evaluated. The method used in each study to assess a measurement property was graded as very good, adequate, doubtful, inadequate, or not applicable. If a study assessed multiple measurement properties, an overall risk-of-bias rating related to the method used was assigned using a worst score counts principle.^21^ For example, if the method used to assess internal consistency in a particular study was rated as adequate and hypothesis testing was rated as very good in the same study, an overall risk-of-bias rating of adequate was assigned for that particular study. Fourth, psychometric measurement properties of PROMs were assessed in individual studies as described in the COSMIN guideline.^16^ These ratings were determined from the results of individual studies and reported as either sufficient, insufficient, inconsistent, or indeterminate.

Fifth, overall performance ratings from all studies evaluating each of the 8 psychometric measurement properties were then rated as either sufficient, insufficient, inconsistent, or indeterminate. If only 1 study assessed a psychometric measurement property, the overall performance rating was equivalent to that reported in the assessment of psychometric measurement properties of PROMs from individual studies because results from only 1 study would be considered. However, if multiple studies assessed the same psychometric measurement property of a PROM, individual ratings from the assessment of psychometric measurement properties of PROMs from individual studies were pooled to provide an overall performance rating of a PROM for that psychometric measurement property. A PROM was deemed to have a sufficient overall rating if more than 50% of individual studies graded it as sufficient (ie, if most studies were in accordance with this finding). If no studies assessed a specific measurement property, the overall rating was reported as indeterminate. For example, if 5 studies assessed internal consistency of a particular PROM and 3 rated it as sufficient, 1 as insufficient, and 1 as indeterminate, an overall rating of sufficient was given because this was the rating for most of the studies. If 1 study rated a measurement property as sufficient and another as insufficient, the measurement property was rated as indeterminate.

Sixth, a level of evidence was assigned for the psychometric measurement properties of each PROM using a modified Grading of Recommendations Assessment, Development, and Evaluation approach^22^ for systematic reviews of clinical trials; the level of evidence was rated as high, moderate, low, or very low depending on (1) risk of bias, (2) inconsistency, (3) imprecision, and (4) indirectness, according to COSMIN recommendations.^16,22^ Studies that failed to report numbers of patients screened or eligible, were missing data or response rates, or had missingness of data from completed PROMs were downgraded for potential risk of bias. Two of us (K.A. and J.O.) independently graded all studies, and conflicts were resolved after discussion with a third one of us (P.S.). Seventh, a summary of the previous 6 steps was used to classify each PROM with a level of recommendation of class A (recommended), B (further research required), or C (not recommended), as described in Table 1.^16,23^

**Table 1.** Summary of Findings

PROM	Content validity (domains, No.)	Psychometric measurement property^a^																Recommendation^c^
		Structural validity		Internal consistency		Cross-cultural validity or measurement invariance		Reliability		Measurement error		Criterion validity		Hypothesis testing		Responsiveness		
		Result	LoE^b^	Result	LoE^b^	Result	LoE^b^	Result	LoE^b^	Result	LoE^b^	Result	LoE^b^	Result	LoE^b^	Result	LoE^b^	
Beck Depression Inventory I	4	?	NA	+	Low	?	NA	?	NA	?	NA	+	Mod	+	Mod	?	NA	B
Beck Depression Inventory II	4	?	Low	+	Low	?	NA	?	NA	?	NA	+	Mod	+	Mod	+	Low	B
Center for Epidemiologic Studies Depression Scale	4	?	NA	?	NA	?	NA	?	NA	?	NA	±	Mod	+	Low	?	Mod	B
Edinburgh Postnatal Depression Scale	3	+	Mod	+	Mod	?	Very low	±	Low	?	NA	+	Mod	+	Mod	+	Mod	A
General Health Questionnaire-12	4	+	Low	?	NA	?	NA	?	Low	?	NA	+	Mod	+	Mod	?	NA	B
Hopkins Symptom Checklist–10	4	?	NA	+	Mod	?	NA	?	Very low	?	NA	?	NA	+	Mod	+	Mod	B
Hospital Anxiety and Depression Scale	4	?	NA	?	NA	?	NA	?	NA	?	NA	?	Low	+	Low	+	Mod	B
Patient Health Questionnaire–9	4	?	NA	?	NA	?	NA	?	NA	?	NA	+	Low	+	Low	?	NA	B
Postpartum Depression Screening Scale	4	-	Mod	+	Mod	?	NA	+	Mod	?	NA	+	Mod	+	Mod	+	Low	B
Zung Self-Rating Depression Scale	4	?	NA	?	NA	?	NA	?	NA	?	NA	?	NA	+	Low	+	Mod	B

Abbreviations: LoE, level of evidence; Mod, moderate; NA, not assessed; PROM, patient-reported outcome measure.

^a^ Ratings for overall quality for each psychometric measurement property are reported as sufficient (+), insufficient (–), inconsistent (±), or indeterminate (?).

^b^ Assessed using Grading of Recommendations Assessment, Development, and Evaluation, reported as high, moderate, low, or very low.

^c^ Recommendation criteria: A indicates evidence for sufficient content validity (any level; ≥3 of 4 domains) and at least low-quality evidence for sufficient internal consistency (which requires sufficient structural validity); these measures can be recommended for use. B indicates measures not categorized as A or C; these measures require further evaluation to assess quality before being recommended for use. C indicates high-quality evidence for a measurement property rated as insufficient; these measures are not recommended for use.

## Results

The Figure summarizes the literature search findings. Among 10 264 postpartum recovery studies screened, 27 PROMs were identified. In total, 43 studies (0.4%) evaluated 10 (37.0%) of the 27 PROMs used in screening for postpartum depression.^24,25,26,27,28,29,30,31,32,33,34,35,36,37,38,39,40,41,42,43,44,45,46,47,48,49,50,51,52,53,54,55,56,57,58,59,60,61,62,63,64,65,66^ The 10 included PROMs were used to screen for postpartum depression in 22 095 women across the studies.

**Figure.**
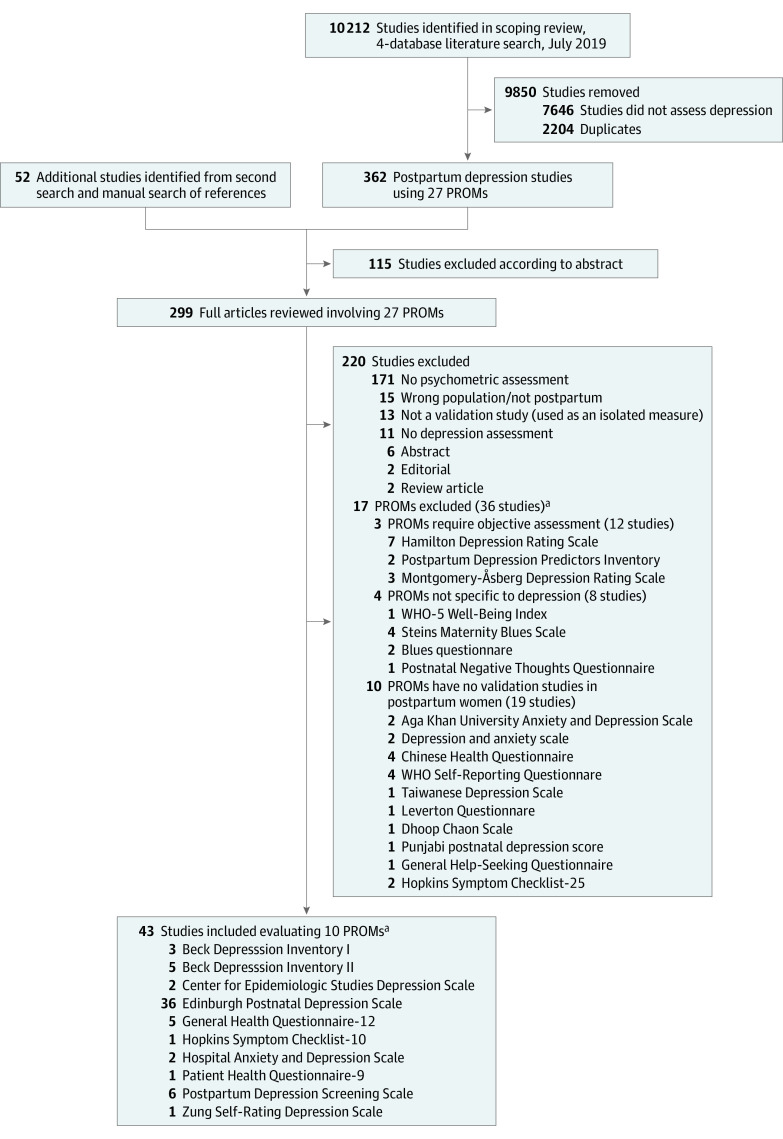
Summary of the Literature Search to Identify Validation Studies for Patient-Reported Outcome Measures (PROMs) of Postpartum Depression WHO indicates World Health Organization. ^a^Some studies evaluated more than 1 PROM used to screen for maternal postpartum depression.

### Study Summary

Table 2 shows the studies^24,25,26,27,28,29,30,31,32,33,34,35,36,37,38,39,40,41,42,43,44,45,46,47,48,49,50,51,52,53,54,55,56,57,58,59,60,61,62,63,64,65,66^ grouped by PROMs. Studies were performed in 18 different languages between 1987 and 2019. The median number of women evaluated in each of the included studies was 223 (range, 43–1453). All studies evaluating psychometric measurement properties of the included PROMs used a prospective study design, and none of the studies were randomized clinical trials. The delivery modes among the participants were not reported in most studies. Depression after operative vaginal delivery was not reported using any of the included PROMs. Two of the 10 PROMs evaluated, the EPDS and the Postpartum Depression Screening Scale, were specifically developed for use with postpartum women. eTable 1 in the Supplement provides a summary of the individual studies, including the languages of the assessed PROMs.

**Table 2.** Summary of Studies Using Each PROM^a^

PROM	Specific obstetric or postpartum population	Validation studies		Postpartum patients studied, range, No.^b^	Language(s) used (No.)	Study years	Birth delivery mode	Postpartum time assessed, range
		No.	Reference No.					
Beck Depression Inventory I	No	3^c^	26, 53, 54	960-1024	English (2), Chinese (1)	1989-2011	All or not stated	3 d to 26 wk
Beck Depression Inventory II	No	5^c^	24, 45, 46, 48, 55	720-1166	English (2), Malay (1), Spanish (1), Taiwanese, (1)	2001-2013	All or not stated	2 wk to 14 mo
Center for Epidemiologic Studies Depression Scale	No	2^c^	56, 57	186-257	English (1), Japanese (1)	2002-2019	All or not stated	1 to 12 mo
Edinburgh Postnatal Depression Scale	Yes	36^c^	24-27, 31-35, 37-45, 47, 50-55, 57-67	13 196-14 044	Arabic (1), Bangladeshi (1), Chinese (2), Danish (1), English (15), Italian (1), Japanese (1), Lithuanian (1), Malay (1), Maltese (1), Norwegian (2), Persian (2), Shona (1), Sinhalese (1), Spanish (3), Taiwanese (1), Thai (1)	1987-2019	All or not stated	3 d to 8 mo
General Health Questionnaire–12	No	5	28, 42, 61, 63, 65	2503^d^	Chinese (1), English (1), Persian (1), Spanish (2)	1998-2019	All or not stated	6 to 28 wk
Hopkins Symptom Checklist–10	No	1	41	223^d^	Arabic (1)	2018	SVD and CD	3 to 8 mo
Hospital Anxiety and Depression Scale	No	2	51, 52	444-454	English (2)	1997-1998	Not stated	4 to 28 wk
Patient Health Questionnaire–9	No	1	60	87-93	English (1)	2008	Not stated	6 to 8 wk
Postpartum Depression Screening Scale	Yes	6	29, 30, 46-49	1838-2119	English (4), Portuguese (1), Spanish (2)	2000-2014	All or not stated	2 wk to 6 mo
Zung Self-Rating Depression Scale	No	1	52	202-212	English (1)	1997	Not stated	4 wk to 8 mo

Abbreviations: CD, cesarean delivery; PROM, patient-reported outcome measure; SVD, spontaneous vaginal delivery.

^a^ All of the studies were prospective.

^b^ Ranges are provided because different numbers of patients were assessed at different time points in the studies.

^c^ Number includes studies that validated more than 1 PROM.

^d^ Total number of patients.

### Content Validity

Table 3 shows the content validity of the included PROMs. All of the 10 included PROMs assessed at least 3 of the 4 proposed domains of postpartum depression. None of the included PROMs had questions about the infant.

**Table 3.** Characteristics and Content Validity of Each Included PROM^a^

PROM	Questions, No.	Period evaluated	Likert scale	Score range	Suggested score cutoff	Freely available	Domains of postpartum depression^b^				Domains, No.
							Affective	Behavioral	Somatic	Interference	
Beck Depression Inventory I	21	7 d	Different for each question	0-63	Normal, 1-10; mild mood disturbance, 11-16; borderline clinical depression, 17-20; moderate depression, 21-30; severe depression, 31-40; extreme depression, >40	Yes	1-12	1, 10, 11, 18	13, 17, 19, 20	14-16, 21	4
Beck Depression Inventory II	21	Past 2 wk	Different for each question	0-63	Minimal depression, 0-13; mild depression, 14-19; moderate depression, 20-28; severe depression, 29-63	No^c^	1-12	1, 10, 11, 18	13, 17, 19, 20	14-16, 21	4
Center for Epidemiologic Studies Depression Scale	20	During past week	Rarely or none of the time (<1 d); some or a little of the time (1-2 d); occasionally or a moderate amount of time (3-4 d); or most of the time (5-7 d)	0-60	Suggestive of need for further evaluation, ≥16	Yes	2-4, 6-9, 14, 17, 18	2, 3, 6, 10, 17, 18	5, 20	15	4
Edinburgh Postnatal Depression Scale	10	Past 7 d	Different for each question	0-30	Possible depression, ≥10	Yes	1-4, 6, 8, 10	4, 5, 9	NA	7	3
General Health Questionnaire–12	12	Past few weeks	4-Point scale: no more than usual, same as usual, less than usual, or much less than usual	0-36	Major depression, 4.5^d^	Yes	3, 5-12	7, 9, 12	1, 4	2	4
Hopkins Symptom Checklist–10	10	Past week	4-Point scale: not at all, a little, quite a bit, or extremely	0-40	Mental distress, mean ≥1.85^e^	Yes	2, 4, 5, 7-10	1, 7	3	6	4
Hospital Anxiety and Depression Scale	14	Past week	Different for each question	0-21	Scored independently for depression and anxiety	Yes	1, 3-5, 7, 8, 10-14	2, 4, 8, 11, 12, 13	2	6	4
Patient Health Questionnaire–9	9	Past 2 wk	4-Point scale: not at all, several days, more than half the days, or nearly every day	0-27	Minimal depression, 1-4; mild depression, 5-9; moderate depression, 10-14; moderately severe depression, 15-19; severe depression, 20-27	Yes	1, 2, 6, 9	2, 5	4, 7, 8	3	4
Postpartum Depression Screening Scale	35	Past 2 wk	5-Point scale ranging from strongly disagree to strongly agree	35-175	Normal adjustment, ≤59; potential symptoms of PPD, 60-79; positive screening result for major PPD, ≥80^f^	No^c^	6, 7, 9, 10, 13-15, 21, 26-34	2, 5, 8-15	16, 19, 20	1, 3, 4	4
Zung Self-Rating Depression Scale	20	Past several days	4-Point scale: a little of the time, some of the time, a good part of the time, or most of the time	20-80	Adult with depressive disorder, index score ≥50 (recommendation to convert the raw score to an index score)	Yes	1, 3, 13-15, 17, 19, 20	3, 5, 13-15	4, 7, 10-12, 16	6	4

Abbreviations: NA, not applicable; PPD, postpartum depression; PROM, patient-reported outcome measure.

^a^ None of the studies had infant items.

^b^ Question numbers stated within domains of postpartum depression.

^c^ Required purchase for use.

^d^ For the General Health Questionnaire–12, Aguado et al^28^ recommend a double test with the Edinburgh Postnatal Depression Scale to increase the sensitivity.

^e^ Questions 1 to 4 relate to anxiety, and 4 to 10 relate to depression symptoms.

^f^ The Postpartum Depression Screening Scale cutoff score of 80 or higher has a sensitivity of 94% and a specificity of 98%.

### Feasibility

Table 3 summarizes PROM availability and associated costs for noncommercial use. The Postpartum Depression Screening Scale and Beck Depression Inventory II are not readily available from links through their associated publications, and there are charges for their use. The PROMs consisted of 9 to 35 questions and evaluated patients during the preceding “several days” to “past few weeks.” The time taken to complete each of the PROMs evaluated was not reported in any of the studies.

eTable 1 in the Supplement shows the response rates (median, 78%; range, 31%-100%). Only 1 of the included studies^30^ reported missingness of data from the completed PROM.

### Risk of Bias

Results of the risk-of-bias assessment of the methods used to evaluate psychometric measurement properties of PROMs in individual studies are provided in eTable 1 in the Supplement. Thirty-three studies^25,26,29,30,32,33,34,35,36,37,38,40,42,43,44,45,46,47,48,49,50,52,54,55,56,57,58,59,60,62,63,64,66^ were graded as very good for methods, 11 studies^24,26,27,28,31,48,51,53,60,61,65^ as adequate, and 2 studies^39,41^ as doubtful (eTable 1 in the Supplement).

### Assessment of Psychometric Measurement Properties of PROMs From Individual Studies and Overall Rating

eTable 2 in the Supplement summarizes the psychometric measurement property ratings for each PROM based on results from individual studies and overall ratings based on pooled results from all studies. None of the included studies assessed measurement error; 1 study^39^ evaluated the property of cross-cultural validity or measurement invariance. Internal consistency was evaluated in 5 of the PROMs (Beck Depression Inventory I, Beck Depression Inventory II, EPDS, Hopkins Symptom Checklist–10, and Postpartum Depression Screening Scale); however, only the EPDS had a sufficient rating for structural validity and internal consistency.

### Level of Evidence

eTable 2 in the Supplement provides a Grading of Recommendations Assessment, Development, and Evaluation level of evidence for the overall rating of each psychometric measurement property for each PROM, with justification for downgrading where applicable. The level of evidence was low or very low for most of the psychometric measurement properties of the PROMs assessed. Studies were frequently downgraded for study design (convenience sampling predisposing to selection bias),^24,27,28,29,30,31,32,35,36,49,51,53,54,60^ inadequate response rate (<60%),^37,44,45,46,55,66^ or low numbers of study patients (<100), resulting in imprecision.^31,33,35,36,57,58,60^

### Summary of Findings

Table 1 summarizes findings from this systematic review. Of the 10 PROMs, the EPDS was the only measure to receive a recommendation level of class A based on adequate content validity, a moderate level of evidence for internal consistency, and sufficient structural validity. Original versions of the Beck Depression Inventory II and the Postpartum Depression Screening Scale were not freely available for use. The remaining PROMs demonstrated sufficient content validity but did not demonstrate sufficient internal consistency and therefore received a class B recommendation. None of the PROMs received a class C recommendation.

## Discussion

The findings suggest that based on the current literature evaluated in this study, the EPDS is the best available PROM to screen for maternal postpartum depression. It was the only PROM in this systematic review that received a recommendation level of class A based on COSMIN criteria.^16^

### Clinical Relevance

To our knowledge, this is the first systematic review to use the COSMIN guideline to assess psychometric measurement properties for validated postpartum depression screening PROMs in the maternal postpartum population. Approximately 1 in 8 women experiences symptoms of postpartum depression,^67^ which is associated with maternal emotional and psychological recovery after childbirth and also with other key aspects of postpartum recovery,^19^ including sleep,^68,69,70,71^ fatigue,^72,73^ maternal-neonatal bonding,^74^ psychosocial support (marital relations, family dysfunction, and social relationships),^75,76^ maternal physical recovery,^77^ and child health.^78,79,80,81^ Postpartum depression is a leading cause of morbidity and mortality.^82^

The EPDS is composed of 10 questions that screen for postpartum depression, resulting in a total score between 0 and 30 (lower scores indicate less postpartum depression). The sensitivity and specificity of the EPDS depend on the cutoff value used. A detailed analysis of 58 studies (including nonobstetric studies) concluded that a cutoff value of 11 or higher maximizes combined sensitivity and specificity of the EPDS.^83^ Among the PROMs evaluated in this analysis, the EPDS was translated into the greatest number of languages (>60) and was used in the greatest number of maternal postpartum studies; however, cross-cultural validity was not adequately evaluated in any of the included studies. Sixteen different versions of the EPDS, including an English language version, were used among the studies that met our inclusion criteria, and sufficient criterion validity (comparison with a gold standard measure) was demonstrated in 10 translated versions, more than any other PROM evaluated. Of importance, the EPDS did not receive an insufficient rating for any psychometric property assessed in this review when adequate or very good methods were used. The EPDS was the most robustly evaluated and best performing PROM in different health care settings, and these findings suggest that the EPDS should be used by clinicians and in future research studies to screen for maternal postpartum depression. Of note, the SCID-5 or psychiatric consultation is the gold standard method for diagnosing maternal postpartum depression. However, these methods require training and expertise and are time consuming and costly, which limit their feasibility for screening large numbers of patients. Ultimately, the decision to use the SCID-5 or EPDS depends on the need for a definitive diagnosis or screening, as determined by individual clinicians and researchers. Further evaluation of psychometric properties of the 9 remaining PROMs would help determine their suitability as alternatives to the EPDS.

PROMs are frequently used in studies to evaluate postpartum recovery health, including postpartum depression. Most studies using PROMs to assess postpartum depression to date have used the EPDS.^11^ The optimal timing for postpartum depression screening remains unclear. Studies included in this review found associations between postpartum depression and maternal well-being,^31^ anxiety and stress,^32^ fatigue,^36^ and insomnia.^37^ Giallo et al^25^ found that postpartum depression and fatigue were distinct but related constructs. Mothers with depressive symptoms in rural Bangladesh were found to be older, to have lower income, and to be less literate and reported more domestic violence and lower emotional bonding with their infants 2 to 3 months postpartum compared with mothers without mental health problems.^38^ Supportive counseling was found by Glavin et al^40^ to be an effective treatment strategy for postpartum depression. Heron et al^66^ reported that hypomanic states were more prevalent postpartum compared with during pregnancy, and Yonkers et al^64^ reported that rates of postpartum depression were similar among Latina and African American women compared with White women.

### Research Implications

Research is frequently performed with measurement instruments of unknown quality, which can be a waste of resources and is potentially unethical.^84,85^ The development of new PROMs can be time consuming and costly. The use of the EPDS may help to maximize efficiency and increase ethicality of future research exploring postpartum depression. By standardizing PROMs used in planned postpartum studies, heterogeneity may be reduced and pooling of data through meta-analysis may be facilitated. Furthermore, the EPDS should be considered for inclusion in any core outcome set developed to evaluate postpartum mental health and recovery after childbirth.

This study highlights the lack of an adequate, freely available PROM that is able to comprehensively assess all postpartum depression domains, and it also identifies gaps in current knowledge relating to the quality assessment of commonly used depression-screening PROMs in the postpartum population. For example, only 1 of the studies in this review assessed cross-cultural validity and measurement invariance with doubtful risk of bias assessment, and none assessed measurement error.

### Limitations

This study has limitations. The authors of the EPDS acknowledge that it contains no items about family relationships or the infant,^86^ and it is also the only PROM in this study that failed to assess all 4 proposed domains of postpartum depression (ie, there was no assessment of somatic symptoms). Of importance, PROMs are not diagnostic tools; they have variable sensitivity and specificity, are dependent on the population, and should only be used to identify women at risk, who require subsequent evaluation and treatment if diagnosed with a mental health disorder.

We acknowledge that the development of author-defined domains of postpartum depression is a subjective process and that the contribution of individual postpartum depression domains may change at different postpartum time points. Postpartum depression is also interconnected with normal hormonal and physiological changes associated with the peripartum period. By gaining consensus among specialists in postpartum recovery (P.S., R.B.G., B.C., and J.C.) and maternal psychological disease (G.L., T.T., and J.C.) and by using a conservative threshold of at least 3 of the 4 proposed domains to demonstrate adequate content validity, we believe that this method allowed us to identify the most appropriate PROMs for screening for postpartum depression.^87^ We also acknowledge that the reporting of findings regarding the quality of individual study methods and results using the COSMIN guideline is subjective and open to differences in opinion. We sought to minimize this variability by grading studies independently by 2 of us and then resolving any conflicts by discussion with a third.

## Conclusions

The findings of this systematic review suggest that the EDPS is currently the best available PROM used to screen for maternal postpartum depression. However, clinicians should be aware of the strengths and weaknesses of PROMs when selecting the most appropriate measure. Additional studies are needed to evaluate the cross-cultural validity, reliability, and measurement error of the EPDS and to further assess psychometric properties of other PROMs to determine their suitability as alternatives.

## Notes

Supplement. **eAppendix 1.** Literature Search Strategy **eAppendix 2.** Proposed Domains of Postpartum Depression **eTable 1.** Summary of Validation Studies for Each Patient-reported Outcome Measure (PROM) of Postnatal Depression **eTable 2.** Risk of Bias Assessment of Methods, Reported Results, Individual and Overall Ratings, and GRADE Level of Evidence From Validation Studies
eReferences
