# Error in Affiliations

**DOI:** 10.1001/jamanetworkopen.2022.29924

**Published:** 2022-08-15

In the Original Investigation titled “Assessment of Patient-Reported Outcome Measures for Maternal Postpartum Depression Using the Consensus-Based Standards for the Selection of Health Measurement Instruments Guideline: A Systematic Review,”^1^ published June 24, 2022, the affiliation for Rania Elkhateb was changed from Library to Department of Anesthesiology at the University of Arkansas for Medical Sciences, Little Rock. This article has been corrected.^1^
