# Supplementary material for: Assessment of Patient-Reported Outcome Measures for Maternal Postpartum Depression Using the Consensus-Based Standards for the Selection of Health Measurement Instruments Guideline: A Systematic Review
Source: JAMA Netw Open. 2022 Jun 24;5(6):e2214885. doi: 10.1001/jamanetworkopen.2022.14885 (PMC9233232; doi:10.1001/jamanetworkopen.2022.14885)
Supplement: Supplement. — eAppendix 1. Literature Search Strategy eAppendix 2. Proposed Domains of Postpartum Depression eTable 1. Summary of Validation Studies for Each Patient-reported Outcome Measure (PROM) of Postnatal Depression eTable 2. Risk of Bias Assessment of Methods, Reported Results, Individual and Overall Ratings, and GRADE Level of Evidence From Validation Studies eReferences [file jamanetwopen-e2214885-s001.pdf]

# Supplementary Online Content

Sultan P, Ando K, Elkhateb R, et al. Assessment of patient-reported outcome measures for maternal postpartum depression using the Consensus-Based Standards for the Selection of Health Measurement Instruments guideline: a systematic review. *JAMA Netw Open*. 2022;5(6):e2214885. doi:10.1001/jamanetworkopen.2022.14885

**eAppendix 1.** Literature Search Strategy
**eAppendix 2.** Proposed Domains of Postpartum Depression
**eTable 1.** Summary of Validation Studies for Each Patient-reported Outcome Measure (PROM) of Postnatal Depression
**eTable 2.** Risk of Bias Assessment of Methods, Reported Results, Individual and Overall Ratings, and GRADE Level of Evidence From Validation Studies
**eReferences**

This supplementary material has been provided by the authors to give readers additional information about their work.

© 2022 Sultan P et al. *JAMA Network Open*.

**eAppendix 1. Literature Search Strategy**

**PubMed – July 1, 2019 – 6,082 results**

("Delivery, Obstetric"[Mesh] OR "Labor, Induced"[Mesh] OR "Extraction, Obstetrical"[Mesh] OR “instrumental delivery” OR “vaginal delivery” OR “vaginal birth” OR “vacuum delivery” OR “vacuum assisted delivery” OR “forceps assisted delivery” OR childbirth) AND ("Postpartum Period"[Mesh] OR "Postnatal Care"[Mesh] OR intrapartum OR postpartum OR postnatal OR "Postoperative Complications"[Mesh] OR "Postoperative Care"[Mesh] OR "Postoperative Period"[Mesh] OR “postoperative” OR “post-operative” or “postsurgery” OR “post surgical” OR “postop” OR recovery OR "Recovery Room"[Mesh] OR "recovery room" OR “post-anesthesia” OR “post-anaesthesia” OR “pacu” OR ((post-anesthesia OR post-anaesthesia) AND acute care unit)) NOT ("Prenatal Care"[Mesh] OR "Prenatal Diagnosis"[Mesh] OR "Prenatal Education"[Mesh] OR prenatal OR antenatal)

AND (("Recovery of Function"[Mesh] OR "Activities of Daily Living"[Mesh] OR "Delayed Emergence from Anesthesia"[Mesh] OR "Fatigue"[Mesh] OR" Anesthesia Recovery Period"[Mesh] OR "Emergence Delirium"[Mesh] OR "Acclimatization"[Mesh] OR "Adaptation, Physiological"[Mesh] OR "Maternal Health"[Mesh] OR “maternal health” OR caretaker OR adaptation OR “fatigue” OR “eat” OR “eating” OR “drink” OR “drinking” OR "Postoperative Nausea and Vomiting"[Mesh] OR "Lethargy"[Mesh] OR nausea OR vomiting))

OR ("Female Urogenital Diseases and Pregnancy Complications"[Mesh] OR "Urinary Incontinence"[Mesh] OR "Uterine Prolapse"[Mesh] OR "Dysmenorrhea"[Mesh] OR "Fecal Incontinence"[Mesh] OR "Gastrointestinal Tract"[Mesh] OR “colorectal” OR “colon” OR rectum OR anus OR urogenital OR urological OR gynecological OR “urinary incontinence” OR “dysmenorrhea” OR “uterine prolapse” OR “fecal incontinence”)

OR ("Pain Management"[Mesh] OR "Pain, Postoperative"[Mesh] OR “pain” OR "Myalgia"[Mesh] OR myalgia OR "Pelvic Pain"[Mesh] OR "Pain"[Mesh] OR “pelvic pain” OR "Headache"[Mesh] OR headache OR "Back Pain"[Mesh] OR “back pain”)

OR (("Mental Health Recovery"[Mesh] OR "Anxiety"[Mesh] OR "Stress Disorders, Post-Traumatic"[Mesh] OR "Depression, Postpartum"[Mesh] OR "Psychotic Disorders"[Mesh] OR "Mood Disorders"[Mesh] OR "Suicidal Ideation"[Mesh] OR "Self-Injurious Behavior"[Mesh] OR "Mental Fatigue"[Mesh] OR "Emotional Adjustment"[Mesh] OR "Pleasure"[Mesh] OR "Happiness"[Mesh] OR "Motivation"[Mesh] OR "Guilt"[Mesh] OR "Social Desirability"[Mesh] OR "Hope"[Mesh] OR "Stress, Psychological"[Mesh] OR "Psychomotor Performance"[Mesh] OR "Appetite"[Mesh] OR "Personal Satisfaction"[Mesh] OR "Adaptation, Psychological"[Mesh] OR "Perception"[Mesh] OR "Bipolar Disorder"[Mesh] OR "Phobic Disorders"[Mesh] OR "Adjustment Disorders"[Mesh] OR "Dissociative Disorders"[Mesh] OR "Dissociative Identity Disorder"[Mesh] OR "Delirium"[Mesh] OR "Neurocognitive Disorders"[Mesh] OR "Cognitive Dysfunction"[Mesh] OR "Behavior"[Mesh] OR "Conduct Disorder"[Mesh] OR "Disruptive, Impulse Control, and Conduct Disorders"[Mesh] OR "Restless Legs Syndrome"[Mesh] OR "Substance-Related Disorders"[Mesh] OR "Personality"[Mesh] OR "Amnesia"[Mesh] OR "Depersonalization"[Mesh] OR "Factitious Disorders"[Mesh] OR "Somatoform Disorders"[Mesh] OR "Conversion Disorder"[Mesh] OR "psychological recovery" OR "mental health recovery" OR

© 2022 Sultan P et al. *JAMA Network Open*.

“mental fatigue” OR "mental health" OR “depression” OR “depressing” OR “depressed” OR anxiety OR anxious OR perpetual psychosis OR “baby blues” OR “mood disorder” OR “self-harm” OR “suicidal ideation” OR “post-traumatic stress disorder” OR “ptsd” OR psychosis OR fatigue OR “emotional” OR (emotion AND control) OR “enjoy” OR enjoyable OR enjoyment OR motivation OR guilt OR wellbeing OR “worth” OR worthless OR worthlessness OR worthy OR “hope” OR hopeful OR hopeless OR hopelessness OR psychomotor OR “self-control” OR appetite OR coping OR perception OR “mania” OR “phobia” OR adjustment OR “reactive” OR dissociative OR “illness anxiety” OR conversion OR factitious OR depersonalization OR amnesia OR somatic OR substance abuse OR “restless leg” OR neurocognitive OR delirium))
OR ("Social Support"[Mesh] OR "Psychosocial Support Systems"[Mesh] OR “social support” OR "Social Adjustment"[Mesh] OR "Social Isolation"[Mesh] OR "Interpersonal Relations"[Mesh] OR “social isolation” OR "Social Adjustment"[Mesh] OR "Social Participation"[Mesh] OR "Social Behavior"[Mesh])
OR ("Sleep"[Mesh] OR “sleep” OR “sleeping” OR “awake” OR insomnia OR narcolepsy OR "Narcolepsy"[Mesh] OR "Sleep Initiation and Maintenance Disorders"[Mesh] OR "Sleep Latency"[Mesh] OR “sleep latency” OR "Sleep Wake Disorders"[Mesh])
OR ("Arousal"[Mesh] OR "Coitus"[Mesh] OR "Sexual Health"[Mesh] OR "Reproductive Health"[Mesh] OR Coitus OR intercourse OR “sexual health” OR "Sexual Dysfunctions, Psychological"[Mesh] OR "Orgasm"[Mesh] OR orgasm OR "Dyspareunia"[Mesh] OR dyspareunia)
OR ("Breast Feeding"[Mesh] OR "Object Attachment"[Mesh] OR "Infant Care"[Mesh] OR “breast feeding” OR bonding OR “infant care”)
AND ((instrumentation[sh] OR methods[sh] OR "Validation Studies"[pt] OR "Comparative Study"[pt] OR "psychometrics"[MeSH] OR psychometr*[tiab] OR clinimetr*[tw] OR clinometr*[tw] OR "outcome assessment (health care)"[MeSH] OR "outcome assessment"[tiab] OR "outcome measure*"[tw] OR "observer variation"[MeSH] OR "observer variation"[tiab] OR "Health Status Indicators"[Mesh] OR "reproducibility of results"[MeSH] OR reproducib*[tiab] OR "discriminant analysis"[MeSH] OR reliab*[tiab] OR unreliab*[tiab] OR valid*[tiab] OR "coefficient of variation"[tiab] OR coefficient[tiab] OR homogeneity[tiab] OR homogeneous[tiab] OR "internal consistency"[tiab] OR (cronbach*[tiab] AND (alpha[tiab] OR alphas[tiab])) OR (item[tiab] AND (correlation*[tiab] OR selection*[tiab] OR reduction*[tiab])) OR agreement[tw] OR precision[tw] OR imprecision[tw] OR "precise values"[tw] OR test-retest[tiab] OR (test[tiab] AND retest[tiab]) OR (reliab*[tiab] AND (test[tiab] OR retest[tiab])) OR stability[tiab] OR interrater[tiab] OR inter-rater[tiab] OR intrarater[tiab] OR intra-rater[tiab] OR intertester[tiab] OR inter-tester[tiab] OR intratester[tiab] OR intra-tester[tiab] OR interobserver[tiab] OR inter-observer[tiab] OR intraobserver[tiab] OR intra-observer[tiab] OR intertechnician[tiab] OR inter-technician[tiab] OR intratechnician[tiab] OR intra-technician[tiab] OR interexaminer[tiab] OR inter-examiner[tiab] OR intraexaminer[tiab] OR intra-examiner[tiab] OR interassay[tiab] OR inter-assay[tiab] OR intraassay[tiab] OR intra-assay[tiab] OR interindividual[tiab] OR inter-individual[tiab] OR intraindividual[tiab] OR intra-individual[tiab] OR interparticipant[tiab] OR inter-participant[tiab] OR intraparticipant[tiab] OR intra-participant[tiab] OR kappa[tiab] OR kappa's[tiab] OR kappas[tiab] OR repeatab*[tw] OR ((replicab*[tw] OR repeated[tw]) AND (measure[tw] OR measures[tw] OR findings[tw] OR result[tw] OR results[tw] OR test[tw] OR tests[tw])) OR

© 2022 Sultan P et al. *JAMA Network Open*.

generaliza*[tiab] OR generalisa*[tiab] OR concordance[tiab] OR (intraclass[tiab] AND correlation*[tiab]) OR discriminative[tiab] OR "known group"[tiab] OR "factor analysis"[tiab] OR "factor analyses"[tiab] OR "factor structure"[tiab] OR "factor structures"[tiab] OR dimension*[tiab] OR subscale*[tiab] OR (multitrait[tiab] AND scaling[tiab] AND (analysis[tiab] OR analyses[tiab])) OR "item discriminant"[tiab] OR "interscale correlation*"[tiab] OR error[tiab] OR errors[tiab] OR "individual variability"[tiab])OR "interval variability"[tiab] OR "rate variability"[tiab] OR (variability[tiab] AND (analysis[tiab] OR values[tiab])) OR (uncertainty[tiab] AND (measurement[tiab] OR measuring[tiab])) OR "standard error of measurement"[tiab] OR sensitiv*[tiab] OR responsive*[tiab] OR (limit[tiab] AND detection[tiab]) OR "minimal detectable concentration"[tiab] OR interpretab*[tiab] OR ((minimal[tiab] OR minimally[tiab] OR clinical[tiab] OR clinically[tiab]) AND (important[tiab] OR significant[tiab] OR detectable[tiab]) AND (change[tiab] OR difference[tiab])) OR (small*[tiab] AND (real[tiab] OR detectable[tiab]) AND (change[tiab] OR difference[tiab])) OR "meaningful change"[tiab] OR "ceiling effect"[tiab] OR "floor effect"[tiab] OR "Item response model"[tiab] OR IRT[tiab] OR Rasch[tiab] OR "Differential item functioning"[tiab] OR DIF[tiab] OR "computer adaptive testing"[tiab] OR "item bank"[tiab] OR "cross-cultural equivalence"[tiab])
NOT (("Delphi Technique"[Mesh] OR "Cross-Sectional Studies"[Mesh] OR "addresses"[Publication Type] OR "biography"[Publication Type] OR "case reports"[Publication Type] OR "comment"[Publication Type] OR "directory"[Publication Type] OR "editorial"[Publication Type] OR "festschrift"[Publication Type] OR "interview"[Publication Type] OR "lectures"[Publication Type] OR "legal cases"[Publication Type] OR "legislation"[Publication Type] OR "letter"[Publication Type] OR "news"[Publication Type] OR "newspaper article"[Publication Type] OR "patient education handout"[Publication Type] OR "popular works"[Publication Type] OR "congresses"[Publication Type] OR "consensus development conference"[Publication Type] OR "consensus development conference, nih"[Publication Type] OR "practice guideline"[Publication Type]) NOT ("animals"[MeSH Terms] NOT "humans"[MeSH Terms]))
Filters: English

**Web of Science – July 1, 2019 – 2,114 results**
("Obstetric Delivery" OR "Induced Labor" OR "Obstetrical Extraction " OR “instrumental delivery” OR “vaginal delivery” OR “vaginal birth” OR “vacuum delivery” OR “vacuum assisted delivery” OR “forceps assisted delivery” OR childbirth) AND ("Postpartum Period" OR "Postnatal Care" OR intrapartum OR postpartum OR postnatal OR "Postoperative Complications" OR "Postoperative Care" OR "Postoperative Period" OR “postoperative” OR “post-operative” or “postsurgery” OR “post surgical” OR “postop” OR recovery OR "recovery room" OR “post-anesthesia” OR “post-anaesthesia” OR “pacu” OR ((post-anesthesia OR post-anaesthesia) AND acute care unit)) NOT ("Prenatal Care" OR "Prenatal Diagnosis" OR "Prenatal Education" OR prenatal OR antenatal)
AND ("Recovery of Function" OR "Activities of Daily Living" OR "Delayed Emergence from Anesthesia" OR "Fatigue" OR "Anesthesia Recovery Period" OR "Emergence Delirium" OR "Acclimatization" OR "Adaptation, Physiological" OR "Maternal Health" OR caretaker OR adaptation OR “fatigue” OR “eat” OR “eating” OR “drink” OR “drinking” OR "Postoperative Nausea and Vomiting" OR "Lethargy"

© 2022 Sultan P et al. *JAMA Network Open*.

OR nausea OR vomiting) OR ("Female Urogenital Diseases and Pregnancy Complications" OR "Urinary Incontinence" OR "Uterine Prolapse" OR "Dysmenorrhea" OR "Fecal Incontinence" OR "Gastrointestinal Tract" OR “colorectal” OR “colon” OR rectum OR anus OR urogenital OR urological OR gynecological OR “urinary incontinence” OR “dysmenorrhea” OR “uterine prolapse” OR “fecal incontinence”) OR ("Pain Management" OR "Pain, Postoperative" OR “pain” OR "Myalgia" OR “pelvic pain” OR "Headache" OR “back pain”) OR ("Mental Health Recovery" OR "Anxiety" OR " Post-Traumatic Stress Disorders" OR "Postpartum Depression" OR "Psychotic Disorders" OR "Mood Disorders" OR "Suicidal Ideation" OR "Self-Injurious Behavior" OR "Mental Fatigue" OR "Emotional Adjustment" OR "Pleasure" OR "Happiness" OR "Motivation" OR "Guilt" OR "Social Desirability" OR "Stress" OR "Psychomotor Performance" OR "Appetite" OR "Personal Satisfaction" OR " Psychological Adaptation" OR "Perception" OR "Bipolar Disorder" OR "Phobic Disorders" OR "Adjustment Disorders" OR "Dissociative Disorders" OR "Dissociative Identity Disorder" OR "Delirium" OR "Neurocognitive Disorders" OR "Cognitive Dysfunction" OR "Behavior" OR "Conduct Disorder" OR "Disruptive, Impulse Control, and Conduct Disorders" OR "Restless Legs Syndrome" OR "Substance-Related Disorders" OR "Personality" OR "Amnesia" OR "Depersonalization" OR "Factitious Disorders" OR "Somatoform Disorders" OR "Conversion Disorder" OR "psychological recovery" OR "mental health" OR “depression” OR “depressing” OR “depressed” OR anxiety OR anxious OR perpetual psychosis OR “baby blues” OR “mood disorder” OR “self-harm” OR “ptsd” OR psychosis OR fatigue OR “emotional” OR (emotion AND control) OR “enjoy” OR enjoyable OR enjoyment OR wellbeing OR “worth” OR worthless OR worthlessness OR worthy OR “hope” OR hopeful OR hopeless OR hopelessness OR psychomotor OR “self-control” OR coping OR perception OR “mania” OR “phobia” OR adjustment OR “reactive” OR dissociative OR “illness anxiety” OR somatic OR neurocognitive OR delirium) OR ("Social Support" OR "Psychosocial Support Systems" OR "Social Adjustment" OR "Social Isolation" OR "Interpersonal Relations" OR “social isolation” OR "Social Adjustment" OR "Social Participation" OR "Social Behavior") OR ("Sleep" OR “sleeping” OR “awake” OR insomnia OR narcolepsy OR "Sleep Initiation and Maintenance Disorders" OR “sleep latency” OR "Sleep Wake Disorders") OR ("Arousal" OR "Coitus" OR "Sexual Health" OR "Reproductive Health" OR Coitus OR intercourse OR "Sexual Dysfunctions" OR orgasm OR dyspareunia) OR ("Breast Feeding" OR "Object Attachment" OR "Infant Care" OR bonding)
AND (((instrumentation OR methods OR "Validation Studies" OR "Comparative Study" OR "psychometrics" OR psychometr* OR clinimetr* OR clinometr* OR "health care outcome assessment" OR "outcome assessment" OR "outcome measure*" OR "observer variation" OR "observer variation" OR "Health Status Indicators" OR "reproducibility of results" OR reproducib* OR "discriminant analysis" OR reliab* OR unreliab* OR valid* OR "coefficient of variation" OR coefficient OR homogeneity OR homogeneous OR "internal consistency" OR (cronbach* AND (alpha OR alphas)) OR (item AND (correlation* OR selection* OR reduction*)) OR agreement OR precision OR imprecision OR "precise values" OR test-retest OR (test AND retest) OR (reliab* AND (test OR retest)) OR stability OR interrater OR inter-rater OR intrarater OR intra-rater OR intertester OR inter-tester OR intratester OR intra-tester OR interobserver OR inter-observer OR intraobserver OR intra-observer OR intertechnician OR inter-technician OR intratechnician OR intra-technician OR interexaminer OR inter-examiner OR intraexaminer OR intra-

© 2022 Sultan P et al. *JAMA Network Open*.

examiner OR interassay OR inter-assay OR intraassay OR intra-assay OR interindividual OR inter-individual OR intraindividual OR intra-individual OR interparticipant OR inter-participant OR intraparticipant OR intra-participant OR kappa OR kappa's OR kappas OR repeatab* OR ((replicab* OR repeated) AND (measure OR measures OR findings OR result OR results OR test OR tests)) OR generaliza* OR generalisa* OR concordance OR (intraclass AND correlation*) OR discriminative OR "known group" OR "factor analysis" OR "factor analyses" OR "factor structure" OR "factor structures" OR dimension* OR subscale* OR (multitrait AND scaling AND (analysis OR analyses)) OR "item discriminant" OR "interscale correlation*" OR error OR errors OR "individual variability") OR "interval variability" OR "rate variability" OR (variability AND (analysis OR values)) OR (uncertainty AND (measurement OR measuring)) OR "standard error of measurement" OR sensitiv* OR responsive* OR (limit AND detection) OR "minimal detectable concentration" OR interpretab* OR ((minimal OR minimally OR clinical OR clinically) AND (important OR significant OR detectable) AND (change OR difference)) OR (small* AND (real OR detectable) AND (change OR difference)) OR "meaningful change" OR "ceiling effect" OR "floor effect" OR "Item response model" OR IRT OR Rasch OR "Differential item functioning" OR DIF OR "computer adaptive testing" OR "item bank" OR "cross-cultural equivalence"))
NOT ((("Delphi Technique" OR "Cross-Sectional Studies" OR "addresses" OR "biography" OR "case reports" OR "comment" OR "directory" OR "editorial" OR "festschrift" OR "interview" OR "lectures" OR "legal cases" OR "legislation" OR "letter" OR "news" OR "newspaper article" OR "patient education handout" OR "popular works" OR "congresses" OR "consensus development conference" OR "nih consensus development conference" OR "practice guideline") NOT ("animals" NOT "humans")))
Filters: English

**CINAHL – July 1, 2019 – 1,035 results**
(MH "Labor, Induced" OR MH "Vacuum Extraction, Obstetrical" OR MH "Delivery, Obstetric" OR MH "Vaginal Birth" OR "Delivery, Obstetric" OR "Labor, Induced" OR "Extraction, Obstetrical" OR “instrumental delivery” OR “vaginal delivery” OR “vaginal birth” OR “vacuum delivery” OR “vacuum assisted delivery” OR “forceps assisted delivery” OR childbirth) AND (MH "Postnatal Care" OR MH "Postnatal Period" OR MH "Postoperative Period" OR MH "Postoperative Care" OR MH "Post Anesthesia Care" OR "Postpartum Period" OR "Postnatal Care" OR intrapartum OR postpartum OR postnatal OR "Postoperative Complications" OR "Postoperative Care" OR "Postoperative Period" OR “postoperative” OR “post-operative” or “postsurgery” OR “post surgical” OR “postop” OR recovery OR "recovery room" OR “post-anesthesia” OR “post-anaesthesia” OR “pacu” OR ((post-anesthesia OR post-anaesthesia) AND acute care unit)) NOT ("Prenatal Care" OR "Prenatal Diagnosis" OR "Prenatal Education" OR prenatal OR antenatal)
AND ((MH "Recovery") OR (MH "Anesthesia Recovery") OR (MH "Activities of Daily Living") OR (MH "Physical Activity") OR (MH "Delirium") OR (MH "Adaptation, Physiological") OR (MH "Adaptation, Psychological") OR (MH "Acclimatization") OR (MH "Nausea") OR (MH "Nausea and Vomiting") OR (MH "Vomiting") OR (MH "Appetite") OR (MH "Fatigue") OR (MH "Mental Fatigue") OR (MH "Muscle Fatigue") OR (MH "Incontinence") OR (MH "Uterine Prolapse") OR (MH "Uterine Hemorrhage") OR (MH "Rectal Prolapse") OR (MH "Uterine

© 2022 Sultan P et al. *JAMA Network Open*.

Inversion") OR (MH "Metrorrhagia") OR (MH "Pelvic Organ Prolapse") OR (MH "Uterine Rupture") OR (MH "Pelvic Pain") OR (MH "Abdominal Pain") OR (MH "Postoperative Pain") OR (MH "Back Pain") OR (MH "Pain") OR (MH "Depression") OR (MH "Depression, Postpartum") OR (MH "Bipolar Disorder") OR (MH "Stress") OR (MH "Stress Disorders, Post-Traumatic") OR (MH "Pleasure") OR (MH "Hope") OR (MH "Optimism") OR (MH "Hopelessness") OR (MH "Self Concept") OR (MH "Personality") OR (MH "Amnesia") OR (MH "Breast Feeding") OR (MH "Mental Health") OR (MH "Sexual Health") OR (MH "Sexual Dysfunction, Female") OR (MH "Coitus") OR (MH "Self-Injurious Behavior") OR (MH "Personal Satisfaction") OR (MH "Sexual Satisfaction") OR (MH "Headache") OR (MH "Muscle Pain") OR (MH "Anxiety") OR (MH "Adjustment Disorders") OR (MH "Dyssomnias") OR (MH "Sleep") OR (MH "Sleep-Wake Transition Disorders") OR (MH "Parasomnias") OR (MH "Sleep Arousal Disorders") OR (MH "Sleep Disorders") OR (MH "Social Behavior Disorders") OR (MH "Social Anxiety Disorders") OR (MH "Social Adjustment") OR (MH "Social Isolation") OR (MH "Parent-Infant Bonding") OR (MH "Infant Care") OR (MH "Perception") OR (MH "Dissociative Disorders") OR (MH "Suicidal Ideation") OR (MH "Happiness") OR (MH "Motivation") OR (MH "Phobic Disorders") OR (MH "Cognition Disorders") OR (MH "Behavior") OR (MH "Parental Behavior") OR (MH "Emotions") OR "Recovery of Function" OR "Activities of Daily Living" OR "Delayed Emergence from Anesthesia" OR "Fatigue" OR "Anesthesia Recovery Period" OR "Emergence Delirium" OR "Acclimatization" OR "Adaptation, Physiological" OR "Maternal Health" OR caretaker OR adaptation OR “fatigue” OR “eat” OR “eating” OR “drink” OR “drinking” OR "Postoperative Nausea and Vomiting" OR "Lethargy" OR nausea OR vomiting) OR ("Female Urogenital Diseases and Pregnancy Complications" OR "Urinary Incontinence" OR "Uterine Prolapse" OR "Dysmenorrhea" OR "Fecal Incontinence" OR "Gastrointestinal Tract" OR “colorectal” OR “colon” OR rectum OR anus OR urogenital OR urological OR gynecological OR “urinary incontinence” OR “dysmenorrhea” OR “uterine prolapse” OR “fecal incontinence”) OR ("Pain Management" OR "Pain, Postoperative" OR “pain” OR "Myalgia" OR “pelvic pain” OR "Headache" OR “back pain”) OR ("Mental Health Recovery" OR "Anxiety" OR " Post-Traumatic Stress Disorders" OR "Postpartum Depression" OR "Psychotic Disorders" OR "Mood Disorders" OR "Suicidal Ideation" OR "Self-Injurious Behavior" OR "Mental Fatigue" OR "Emotional Adjustment" OR "Pleasure" OR "Happiness" OR "Motivation" OR "Guilt" OR "Social Desirability" OR "Stress" OR "Psychomotor Performance" OR "Appetite" OR "Personal Satisfaction" OR " Psychological Adaptation" OR "Perception" OR "Bipolar Disorder" OR "Phobic Disorders" OR "Adjustment Disorders" OR "Dissociative Disorders" OR "Dissociative Identity Disorder" OR "Delirium" OR "Neurocognitive Disorders" OR "Cognitive Dysfunction" OR "Behavior" OR "Conduct Disorder" OR "Disruptive, Impulse Control, and Conduct Disorders" OR "Restless Legs Syndrome" OR "Substance-Related Disorders" OR "Personality" OR "Amnesia" OR "Depersonalization" OR "Factitious Disorders" OR "Somatoform Disorders" OR "Conversion Disorder" OR "psychological recovery" OR "mental health" OR “depression” OR “depressing” OR “depressed” OR anxiety OR anxious OR perpetual psychosis OR “baby blues” OR “mood disorder” OR “self-harm” OR “ptsd” OR psychosis OR fatigue OR “emotional” OR (emotion AND control) OR “enjoy” OR enjoyable OR enjoyment OR wellbeing OR “worth” OR worthless OR worthlessness OR worthy OR “hope” OR hopeful OR hopeless OR hopelessness OR psychomotor OR “self-control” OR coping OR perception OR “mania” OR “phobia” OR

adjustment OR “reactive” OR dissociative OR “illness anxiety” OR somatic OR neurocognitive OR delirium) OR ("Social Support" OR "Psychosocial Support Systems" OR "Social Adjustment" OR "Social Isolation" OR "Interpersonal Relations" OR “social isolation” OR "Social Adjustment" OR "Social Participation" OR "Social Behavior") OR ("Sleep" OR “sleeping” OR “awake” OR insomnia OR narcolepsy OR "Sleep Initiation and Maintenance Disorders" OR “sleep latency” OR "Sleep Wake Disorders") OR ("Arousal" OR "Coitus" OR "Sexual Health" OR "Reproductive Health" OR Coitus OR intercourse OR "Sexual Dysfunctions" OR orgasm OR dyspareunia) OR ("Breast Feeding" OR "Object Attachment" OR "Infant Care" OR bonding)
AND (((instrumentation OR methods OR "Validation Studies" OR "Comparative Study" OR "psychometrics" OR psychometr* OR clinimetr* OR clinometr* OR "health care outcome assessment" OR "outcome assessment" OR "outcome measure*" OR "observer variation" OR "observer variation" OR "Health Status Indicators" OR "reproducibility of results" OR reproducib* OR "discriminant analysis" OR reliab* OR unreliab* OR valid* OR "coefficient of variation" OR coefficient OR homogeneity OR homogeneous OR "internal consistency" OR (cronbach* AND (alpha OR alphas)) OR (item AND (correlation* OR selection* OR reduction*)) OR agreement OR precision OR imprecision OR "precise values" OR test-retest OR (test AND retest) OR (reliab* AND (test OR retest)) OR stability OR interrater OR inter-rater OR intrarater OR intra-rater OR intertester OR inter-tester OR intratester OR intra-tester OR interobserver OR inter-observer OR intraobserver OR intra-observer OR intertechnician OR inter-technician OR intratechnician OR intra-technician OR interexaminer OR inter-examiner OR intraexaminer OR intra-examiner OR interassay OR inter-assay OR intraassay OR intra-assay OR interindividual OR inter-individual OR intraindividual OR intra-individual OR interparticipant OR inter-participant OR intraparticipant OR intra-participant OR kappa OR kappa's OR kappas OR repeatab* OR ((replicab* OR repeated) AND (measure OR measures OR findings OR result OR results OR test OR tests)) OR generaliza* OR generalisa* OR concordance OR (intraclass AND correlation*) OR discriminative OR "known group" OR "factor analysis" OR "factor analyses" OR "factor structure" OR "factor structures" OR dimension* OR subscale* OR (multitrait AND scaling AND (analysis OR analyses)) OR "item discriminant" OR "interscale correlation*" OR error OR errors OR "individual variability") OR "interval variability" OR "rate variability" OR (variability AND (analysis OR values)) OR (uncertainty AND (measurement OR measuring)) OR "standard error of measurement" OR sensitiv* OR responsive* OR (limit AND detection) OR "minimal detectable concentration" OR interpretab* OR ((minimal OR minimally OR clinical OR clinically) AND (important OR significant OR detectable) AND (change OR difference)) OR (small* AND (real OR detectable) AND (change OR difference)) OR "meaningful change" OR "ceiling effect" OR "floor effect" OR "Item response model" OR IRT OR Rasch OR "Differential item functioning" OR DIF OR "computer adaptive testing" OR "item bank" OR "cross-cultural equivalence"))
NOT ((("Delphi Technique" OR "Cross-Sectional Studies" OR "addresses" OR "biography" OR "case reports" OR "comment" OR "directory" OR "editorial" OR "festschrift" OR "interview" OR "lectures" OR "legal cases" OR "legislation" OR "letter" OR "news" OR "newspaper article" OR "patient education handout" OR "popular works" OR "congresses" OR "consensus development conference" OR "nih consensus development conference" OR "practice guideline") NOT ("animals" NOT "humans")))

© 2022 Sultan P et al. *JAMA Network Open*.

Filters: English, Academic Journals and Dissertations

**EMBASE – July 1, 2019 – 981 results**
('delivery, obstetric'/de OR 'labor, induced'/de OR 'extraction, obstetrical'/de OR 'instrumental delivery'/de OR 'vaginal delivery'/de OR 'vaginal birth'/de OR 'vacuum delivery'/de OR 'vacuum assisted delivery'/de OR 'forceps assisted delivery' OR 'childbirth'/de) AND ('postpartum period'/de OR 'postnatal care'/de OR intrapartum OR 'postpartum'/de OR postnatal OR 'postoperative complications'/de OR 'postoperative care'/de OR 'postoperative period'/de OR 'postoperative' OR 'post-operative' OR 'postsurgery' OR 'post surgical' OR 'postop' OR 'recovery'/de OR 'recovery room'/de OR 'post-anesthesia' OR 'post-anaesthesia' OR 'pacu' OR (('post anesthesia' OR 'post anaesthesia') AND acute AND 'care'/de AND 'unit'/de))
AND 'recovery of function'/de OR 'activities of daily living'/de OR 'delayed emergence from anesthesia'/de OR 'anesthesia recovery period'/de OR 'emergence delirium'/de OR 'acclimatization'/de OR 'adaptation, physiological'/de OR 'maternal health'/de OR caretaker OR 'adaptation'/de OR 'eat' OR 'eating'/de OR 'drink' OR 'drinking'/de OR 'postoperative nausea and vomiting'/de OR 'lethargy'/de OR 'nausea'/de OR 'vomiting'/de OR 'female urogenital diseases and pregnancy complications'/de OR 'gastrointestinal tract'/de OR 'colorectal' OR 'colon'/de OR 'rectum'/de OR 'anus'/de OR urogenital OR urological OR gynecological OR 'urinary incontinence'/de OR 'dysmenorrhea'/de OR 'uterine prolapse'/de OR 'fecal incontinence'/de OR 'pain management'/de OR 'pain, postoperative'/de OR 'pain'/de OR 'myalgia'/de OR 'pelvic pain'/de OR 'headache'/de OR 'back pain'/de OR (('mental health recovery'/de OR 'post-traumatic stress disorders' OR 'postpartum depression'/de OR 'psychotic disorders'/de OR 'mood disorders'/de OR 'suicidal ideation'/de OR 'self-injurious behavior'/de OR 'mental fatigue'/de OR 'emotional adjustment'/de OR 'pleasure'/de OR 'happiness'/de OR 'motivation'/de OR 'guilt'/de OR 'social desirability'/de OR 'stress'/de OR 'psychomotor performance'/de OR 'appetite'/de OR 'personal satisfaction'/de OR 'psychological adaptation'/de OR 'perception'/de OR 'bipolar disorder'/de OR 'phobic disorders'/de OR 'adjustment disorders'/de OR 'dissociative disorders'/de OR 'dissociative identity disorder'/de OR 'delirium'/de OR 'neurocognitive disorders'/de OR 'cognitive dysfunction'/de OR 'behavior'/de OR 'conduct disorder'/de OR 'disruptive, impulse control, and conduct disorders'/de OR 'restless legs syndrome'/de OR 'substance-related disorders'/de OR 'personality'/de OR 'amnesia'/de OR 'depersonalization'/de OR 'factitious disorders'/de OR 'somatoform disorders'/de OR 'conversion disorder'/de OR 'psychological recovery' OR 'mental health'/de OR 'depression'/de OR 'depressing' OR 'depressed' OR 'anxiety'/de OR anxious OR perpetual) AND 'psychosis'/de) OR 'baby blues' OR 'mood disorder'/de OR 'self-harm'/de OR 'ptsd'/de OR 'psychosis'/de OR 'fatigue'/de OR 'emotional' OR ('emotion'/de AND 'control'/de) OR 'enjoy' OR enjoyable OR 'enjoyment'/de OR 'wellbeing'/de OR 'worth' OR worthless OR 'worthlessness'/de OR worthy OR 'hope'/de OR hopeful OR hopeless OR 'hopelessness'/de OR psychomotor OR 'self-control'/de OR 'coping'/de OR 'perception'/de OR 'mania'/de OR 'phobia'/de OR 'adjustment'/de OR 'reactive' OR dissociative OR 'illness anxiety' OR somatic OR neurocognitive OR 'delirium'/de OR 'social support'/de OR 'psychosocial support systems'/de OR 'interpersonal relations'/de OR 'social isolation'/de OR 'social adjustment'/de OR 'social participation'/de OR 'social behavior'/de OR 'sleep'/de OR 'sleeping'/de OR 'awake'/de OR 'insomnia'/de OR 'narcolepsy'/de OR 'sleep initiation and maintenance disorders'/de OR 'sleep latency'/de OR 'sleep wake disorders'/de OR

© 2022 Sultan P et al. *JAMA Network Open*.

'arousal'/de OR 'sexual health'/de OR 'reproductive health'/de OR 'coitus'/de OR 'intercourse'/de OR 'sexual dysfunctions' OR 'orgasm'/de OR 'dyspareunia'/de OR 'breast feeding'/de OR 'object attachment'/de OR 'infant care'/de OR 'bonding'/de
AND ('instrumentation'/de OR 'methods'/de OR 'validation studies'/de OR 'comparative study'/de OR 'psychometrics'/de OR psychometr* OR clinimetr* OR clinometr* OR 'health care outcome assessment' OR 'outcome assessment'/de OR 'outcome measure*' OR 'observer variation'/de OR 'health status indicators'/de OR 'reproducibility of results'/de OR reproducib* OR 'discriminant analysis'/de OR reliab* OR unreliab* OR valid* OR 'coefficient of variation'/de OR coefficient OR 'homogeneity'/de OR homogeneous OR 'internal consistency'/de OR (cronbach* AND (alpha OR alphas)) OR (item AND (correlation* OR selection* OR reduction*)) OR 'agreement'/de OR 'precision'/de OR imprecision OR 'precise values' OR 'test retest' OR ('test'/de AND retest) OR (reliab* AND ('test'/de OR retest)) OR 'stability'/de OR interrater OR 'inter rater' OR intrarater OR 'intra rater' OR intertester OR 'inter tester' OR intratester OR 'intra tester' OR interobserver OR 'inter observer' OR intraobserver OR 'intra observer' OR intertechnician OR 'inter technician' OR intratechnician OR 'intra technician' OR interexaminer OR 'inter examiner' OR intraexaminer OR 'intra examiner' OR interassay OR 'inter assay' OR intraassay OR 'intra assay' OR interindividual OR 'inter individual' OR intraindividual OR 'intra individual' OR interparticipant OR 'inter participant' OR intraparticipant OR 'intra participant' OR kappa OR kappas OR repeatab* OR ((replicab* OR repeated) AND (measure OR measures OR findings OR result OR results OR 'test'/de OR tests)) OR generaliza* OR generalisa* OR 'concordance'/de OR (intraclass AND correlation*) OR discriminative OR 'known group' OR 'factor analysis'/de OR 'factor analyses' OR 'factor structure'/de OR 'factor structures' OR dimension* OR subscale* OR (multitrait AND 'scaling'/de AND ('analysis'/de OR analyses)) OR 'item discriminant' OR 'interscale correlation*' OR 'error'/de OR errors OR 'individual variability' OR 'interval variability' OR 'rate variability' OR ('variability'/de AND ('analysis'/de OR values)) OR ('uncertainty'/de AND ('measurement'/de OR measuring)) OR 'standard error of measurement'/de OR sensitiv* OR responsive* OR (limit AND 'detection'/de) OR 'minimal detectable concentration' OR interpretab* OR ((minimal OR minimally OR 'clinical'/de OR clinically) AND (important OR significant OR detectable) AND ('change'/de OR difference)) OR (small* AND (real OR detectable) AND ('change'/de OR difference)) OR 'meaningful change' OR 'ceiling effect'/de OR 'floor effect'/de OR 'item response model' OR irt OR rasch OR 'differential item functioning'/de OR dif OR 'computer adaptive testing'/de OR 'item bank' OR 'cross-cultural equivalence')
NOT (('delphi technique'/de OR 'cross-sectional studies'/de OR 'addresses' OR 'biography'/de OR 'case reports' OR 'comment' OR 'directory'/de OR 'editorial'/de OR 'festschrift' OR 'interview'/de OR 'lectures' OR 'legal cases' OR 'legislation'/de OR 'letter'/de OR 'news' OR 'newspaper article' OR 'patient education handout' OR 'popular works' OR 'congresses'/de OR 'consensus development conference'/de OR 'nih consensus development conference' OR 'practice guideline'/de OR NOT ("Prenatal Care" OR "Prenatal Diagnosis" OR "Prenatal Education" OR prenatal OR antenatal OR 'animals'/de))
Filters: English

**Total = 10,212**
**Endnote duplicate and animal removal = 8,585**
**Rayyan duplicate removal = 8,008**

© 2022 Sultan P et al. *JAMA Network Open*.

## eAppendix 2. Proposed domains of postpartum depression

| Affective | Behavioral | Somatic | Interference |
|---|---|---|---|
| Low mood / sad / happiness / depressed / blue / enjoyment | | Weight (increase / decrease) | Work |
| Anxiety | | Psychomotor agitation / retardation (decreased physical movement) | Relationships |
| Anger | | Fatigue<br>Loss of energy / tiredness | Social functioning |
| Irritability | | Slow thought process /alertness | Looks / appearance |
| Restlessness / calmness | | Reduced concentration / indecisiveness / muddled / forgetful / focus | Maternal-neonatal bonding |
| Emotional lability / emotional numbness / over emotional | | Aches / pains, upset stomach constipation | Sexual function |
| Worthlessness / inappropriate guilt / blame (may be delusional) | Tearfulness | Changes in taste/food does not taste good | Breastfeeding |
| Tense | Scared / panicky / fear | Weak | Ability to care for self |
| Helplessness | Able to laugh | | Ability to care for child |
| Hopelessness | Appetite (Increase / decrease) | | Sleep |
| Stress | Self-harm behaviors | | |
| Loneliness | | | |
| Desire to be alone | | | |
| Overwhelmed | | | |
| Overly sensitive | | | |
| Loss of interest / pleasure | | | |
| Difficult to show feelings | | | |
| Brooding on things | | | |
| Self-esteem / lack of confidence | | | |
| Feel like a failure / letting everyone down | | | |
| Feel everything is a burden / effort | | | |
| Feel like being punished | | | |
| Self-disappointment / feeling sorry for self | | | |
| Cannot overcome difficulties / face up to problems | | | |
| Thoughts of self-harm | | | |
| Suicidal ideation /<br>Thoughts of death | | | |
| Thoughts of harming the baby | | | |

**Affective**- a subjective feeling or thought process (all items can be preceded by: “I feel….”)
**Behavioral**- a behavior which can easily be seen objectively by another person
**Somatic**- feelings which have a physical manifestation or consequence
**Interference** – a patient’s psychological state is interfering with different aspects of functioning

**Assessment of Content Validity:** Adequate content validity can be considered for a patient-reported outcome measure if ≥75% (≥3) of the above domains are assessed using any given measure

The following 2 PROM items were felt to not be consistent with a diagnosis postpartum depression and are not present in the above table:

- dissociation (Hopkins Symptom Checklist-10);
- faintness / dizziness (Hopkins Symptom Checklist-10)

© 2022 Sultan P et al. *JAMA Network Open*.

## eTable 1. Summary of validation studies for each patient-reported outcome measures (PROMs) of postnatal depression

| First Author | Year | Country of Study | Language of instrument | Patient number (n) | Response Rate % | Methodology |
|---|---|---|---|---|---|---|
| **Beck Depression Inventory I** | | | | | | |
| Harris[a] | 1989 | UK | English | 126-129 | 86-88 | Adequate |
| Ji[a] | 2011 | USA | English | 534 | 75 | Very good |
| Lau[a] | 2010 | China | Chinese | 300-343 | Not stated | Very good |
| **Beck Depression Inventory II** | | | | | | |
| Beck[a] | 2001 | USA | English | 150 | Not stated | Very good |
| Chaudron[a] | 2010 | USA | English | 198 | 31 | Very good |
| Lara[a] | 2013 | Mexico | Spanish | 136 | 36 | Very good |
| Teng[a] | 2005 | Taiwan, China | Taiwanese | 175-328 | 53-82 | Very good |
| Wan Mahmud[a] | 2004 | Malaysia | Malay | 61-354 | Not stated | Adequate |
| **Center for Epidemiologic Studies Depression Scale** | | | | | | |
| Beeghly | 2002 | USA | English | 106-177 | 60 | Very good |
| Sasaki[a] | 2019 | Japan | Japanese | 80 | 93 | Very good |
| **Edinburgh Postnatal Depression Scale** | | | | | | |
| Alvarado-Esquivel | 2006 | Mexico | Spanish | 49-51 | Not stated | Very good |
| Bassi | 2017 | Italy | Italian | 81 | 98 | Adequate |
| Beck[a] | 2001 | USA | English | 150 | Not stated | Very good |
| Bunevicius | 2009 | Lithuania | Lithuanian | 94 | Not stated | Very good |
| Chaudron[a] | 2010 | USA | English | 198 | 31 | Very good |
| Chibanda | 2010 | Zimbabwe | Shona | 210 | 94 | Very good |
| Coo | 2015 | Australia | English | 96 -101 | 78-82 | Very good |
| Condon[a] | 1997 | Australia | English | 202-212 | 78-82 | Very good |
| Cox | 1987 | UK | English | 84 | Not stated | Very good |
| Doering Runquist | 2009 | USA | English | 43 | 63 | Very good |
| Dorheim | 2014 | Norway | Norwegian | 2386 | 51 | Very good |
| Edhborg | 2011 | Bangladesh | Bangladeshi | 672 | 93 | Very good |
| Felice | 2005 | Malta | Maltese | 223 | 93 | Doubtful |
| Garcia-Esteve | 2003 | Spain | Spanish | 1201 | Not stated | Very good |
| Giallo | 2015 | Australia | English | 1099 - 1390 | 73-92 | Very good |
| Glavin | 2010 | Norway | Norwegian | 146 - 186 | 64-82 | Very good |
| Hanusa[a] | 2008 | USA | English | 87- 93 | 70-76 | Very good |
| Harris[a] | 1989 | UK | English | 126-129 | 86-88 | Adequate |
| Heron | 2009 | UK | English | 203-207 | 59-60 | Very good |
| Ji[a] | 2011 | USA | English | 534 | 75 | Very good |
| Khalifa[a] | 2018 | Sudan | Arabic | 223 | 74 | Doubtful |
| Lau[a] | 2010 | China | Chinese | 300-343 | Not stated | Adequate |
| Lee[a] | 2000 | China | Chinese | 145 | 66 | Adequate |
| Mazhari[a] | 2007 | Iran | Persian | 200 | Not stated | Very good |
| Montezeri | 2007 | Iran | Persian | 100 | Not stated | Very good |
| Murray | 1990 | UK | English | 674 | 96 | Very good |
| Mwape[a] | 2019 | Zambia | English | 300 | Not stated | Adequate |
| Navarro[a] | 2007 | Spain | Spanish | 405 | 95 | Very good |
| Pitanupong | 2007 | Thailand | Thai | 351 | 57 | Very good |

© 2022 Sultan P et al. *JAMA Network Open*.

| | | | | | | |
|---|---|---|---|---|---|---|
| Rowel | 2008 | Sri Lanka | Sinhalese | 204 | Not stated | Very good |
| Sasaki[a] | 2019 | Japan | Japanese | 80 | 93 | Very good |
| Smith-Nielsen | 2018 | Denmark | Danish | 320 | 91 | Adequate |
| Teng[a] | 2005 | Taiwan, China | Taiwanese | 175-328 | 53-82 | Very good |
| Thompson[a] | 1998 | UK | English | 242 | 78 | Adequate |
| Wan Mahmud[a] | 2004 | Malyasia | Malay | 61-354 | Not stated | Very good |
| Yonkers | 2001 | USA | English | 802 | 90 | Very good |
| **General Health Questionnaire-12** | | | | | | |
| Aguado | 2012 | Spain | Spanish | 1453 | Not stated | Adequate |
| Lee[a] | 2000 | China | Chinese | 145 | 66 | Very good |
| Mazhari [a] | 2007 | Iran | Persian | 200 | Not stated | Very good |
| Mwape[a] | 2019 | Zambia | English | 300 | Not stated | Adequate |
| Navarro[a] | 2007 | Spain | Spanish | 405 | 95 | Very good |
| **Hopkins Symptom Checklist-10** | | | | | | |
| Khalifa[a] | 2018 | Sudan | Arabic | 223 | 74 | Doubtful |
| **Hospital Anxiety and Depression Scale** | | | | | | |
| Condon[a] | 1997 | Australia | English | 202-212 | 78-82 | Very good |
| Thompson[a] | 1998 | UK | English | 242 | 78 | Adequate |
| **Patient Health Questionnnaire-9** | | | | | | |
| Hanusa[a] | 2008 | USA | English | 87- 93 | 71-76 | Adequate |
| **Postpartum Depression Screening Scale** | | | | | | |
| Beck | 2000 | USA | English | 525 | 100 | Very good |
| Beck | 2003 | USA | Spanish | 377 | Not stated | Very good |
| Beck[a] | 2001 | USA | English | 150 | Not stated | Adequate |
| Blucker | 2014 | USA | English | 110- 385 | 100 | Very good |
| Lara[a] | 2013 | Mexico | Spanish | 136 | 36 | Very good |
| Pereira | 2013 | Portugal | Portuguese | 453 | 93 | Very good |
| **Zung Self Rating Depression Scale** | | | | | | |
| Condon[a] | 1997 | Australia | English | 202-212 | 78-82 | Very good |

a= includes studies evaluating >1 validated PROM used to screen for maternal postpartum depression; response rates presented as a range where different groups or time points within the study reported; methodology reported according to the COSMIN risk of bias checklist using a 'worst score counts' principle and evaluated related to measurement property assessed.

© 2022 Sultan P et al. *JAMA Network Open*.

**eTable 2. Risk of bias assessment of methods, reported results, individual and overall ratings and GRADE level of evidence from validation studies**

| Instrument | Study Author, Year | Methods (risk of bias) Assessment | Individual results reported in included studies for each psychometric property | Rating of individual results | Overall rating | GRADE level of evidence |
|---|---|---|---|---|---|---|
| **Structural validity** | | | | | | |
| Beck Depression Inventory I | | Not assessed | Not assessed | ? | ? | NA |
| Beck Depression Inventory II | Wan Mahmud,[a] 2004[1] | Adequate | Exploratory factor analysis using varimax rotational methods | ? | ? | Low<br>(Adequate sample size, but unknown response rate) |
| Center for Epidemiologic Studies Depression Scale | | Not assessed | Not assessed | ? | ? | NA |
| Edinburgh Postnatal Depression Score | Giallo, 2015[2] | Very good | At 6 months: $\chi2$ (35, n=1390) = 391.88, p<0.001, RMSEA=0.09, TLI=0.96, CFI=0.97;<br>At 4 years $\chi2$ (35, n=1099)=273.93, p<0.001, RMSEA=0.08, TLI=0.98, CFI=0.99 | + | + | Moderate<br>(Convenience sampling, adequate sample size, missing data not reported. eligibility/ response rates not reported in 1 study[3]) |
| | Lau,[a] 2010[3] | Very good | SRMR=0.0516, RMSEA=0.048, CFI=0.991, p<0.001 | + | | |
| | Smith-Nielsen, 2019[4] | Adequate | Exploratory factor analysis employing the oblique rotation (promax) | ? | | |
| General Health Questionnaire-12 | Aguado, 2012[5] | Very good | CFA=0.048, RMSEA CFI=0.990 | + | + | Low<br>(Convenience sampling from another study, unknown response rate) |
| Hopkins Symptom Checklist-10 | | Not assessed | Not assessed | ? | ? | NA |
| Hospital Anxiety and Depression Scale | | Not assessed | Not assessed | ? | ? | NA |
| Patient Health Questionnnaire-9 | | Not assessed | Not assessed | ? | ? | NA |
| Postpartum Depression Screening Scale | Beck, 2003[6] | Very good | CFI=0.80, RMSEA=0.089, SRMR=0.066 | - | - | Moderate<br>(Convenience sampling, adequate sample size in both studies. 100% response rate. Missing data not known,[6] 1 patient removed[7]) |
| | Blucker, 2014[7] | Very good | 5-factor model had reasonable fit: $\chi2$=1339.70 (550); P<0.01; CFI=0.85; root RMSEA=0.06; 90% CI=0.058 to 0.067.<br>In study 2, CFA was used to test the construct validity or goodness of fit of the 7-factor and 5-factor models. Results for the 7-factor model indicated unacceptable fit: $\chi2$ (539)=959.10; P<0.01; CFI=0.76; and RMSEA=0.09. The 5-factor model was also poor: $\chi2$ (550)=992.95; P<0.01; CFI=0.75; and RMSEA=0.09 | - | | |
| Zung Depression scale | | Not assessed | Not assessed | ? | ? | NA |

© 2022 Sultan P et al. *JAMA Network Open*.

| Internal consistency | | | | | | |
|---|---|---|---|---|---|---|
| Beck Depression Inventory I | Lau,[a] 2010[3] | Very good | Cronbach's α=0.87 | + | + | Low<br>(Adequate sample size but unknown response rate) |
| Beck Depression Inventory II | Wan Mahmud,[a] 2004[1] | Very good | Cronbach's α =0.89 | + | + | Low<br>(Adequate sample size but unknown response rate) |
| Center for Epidemiologic Studies Depression Scale | | Not assessed | Not assessed | ? | ? | NA |
| Edinburgh Postnatal Depression Score | Bassi, 2017[8] | Very good | Cronbach's α=0.79 | + | + | Moderate<br>(Convenience sampling with Low (3 studies[8,9 10]) and Very low sample size (1 study[11])<br>Response rate unknown (3 studies[3,9,12]) |
| | Bunevicius, 2009[13] | Very good | Cronbach's α=0.83 | + | | |
| | Chibanda, 2010[14] | Very good | Cronbach's α=0.87 | + | | |
| | Coo, 2015[10] | Very good | Cronbach's α at 7-10 days and 10-12 weeks were 0.84, and 0.88, respectively | + | | |
| | Cox, 1987[9] | Very good | Standardized α -coefficient 0.87 | + | | |
| | Doering Runquist, 2009[11] | Very good | Cronbach's α: 1 month=0.89, 3 months=0.90, and 6 month=0.87 | + | | |
| | Dorheim, 2014[15] | Very good | Cronbach's α=0.86 at 8 weeks | + | | |
| | Edhborg, 2011[16] | Very good | Cronbach's α=0.70 | + | | |
| | Felice, 2005[17] | Very good | Cronbach's α=0.98 | + | | |
| | Giallo, 2015[2] | Very good | Cronbach's α=0.85 at 6 months | + | | |
| | Glavin, 2010[18] | Very good | Cronbach's α=0.81 | + | | |
| | Khalifa,[a] 2018[19] | Very good | Cronbach's α=0.83 | + | | |
| | Lau,[a] 2010[3] | Very good | Cronbach's α=0.78 | + | | |
| | Mazhari,[a] 2007[20] | Very good | Cronbach's α=0.83 | + | | |
| | Montazeri, 2007[12] | Very good | Cronbach's α=0. 77 at T1, 0.86 at T2 | + | | |
| | Pitanupong, 2007[21] | Very good | Internal consistency=0.8 | + | | |
| | Smith-Nielsen, 2018[4] | Very good | Cronbach's α=0.822 | + | | |
| | Teng,[a] 2005[22] | Very good | Cronbach's α=0.87 | + | | |
| General Health Questionnaire-12 | | Not assessed | Not assessed | ? | ? | NA |
| Hopkins Symptom Checklist-10 | Khalifa,[a] 2018[19] | Very good | Internal consistency=0.77 | + | + | Moderate<br>(Adequate sample size and response rate. No missing data reported) |
| Hospital Anxiety and Depression Scale | | Not assessed | ? | ? | ? | N/A |
| Patient Health Questionnnaire-9 | | Not assessed | ? | ? | ? | N/A |
| Postpartum Depression Screening Scale | Beck, 2003[6] | Very good | Cronbach's α=0.95 total + [0.94 (Mexican), 0.96 (Puerto Rican), 0.93 (other)] | + | + | Moderate<br>(Good sample size, response rate 36%[23], missing data only reported in 1 study[7]) |
| | Beck, 2000[24] | Very good | α internal consistency reliabilities ranged from 0.83 (sleeping/eating disturbances) to 0.94 (loss of self). | + | | |
| | Beck,[a] 2001[25] | Very good | Cronbach's α=0.8 to 0.91 | + | | |
| | Blucker, 2014[7] | Very good | Internal consistency=0.95 | + | | |
| | Lara,[a] 2013[23] | Very good | Cronbach's α=0.96 for T1 and T2 | + | | |
| | Pereira,[a] 2013[26] | Very good | Cronbach's α=0.96 | + | | |
| Zung Depression scale | Not assessed | Not assessed | Not assessed | ? | ? | NA |

© 2022 Sultan P et al. *JAMA Network Open*.

| | | | | | | |
|---|---|---|---|---|---|---|
| **Cross cultural validity/ Measurement invariance** | | | | | | |
| Beck Depression Inventory I | | Not assessed | Not assessed | ? | ? | NA |
| Beck Depression Inventory II | | Not assessed | Not assessed | ? | ? | NA |
| Center for Epidemiologic Studies Depression Scale | | Not assessed | Not assessed | ? | ? | NA |
| Edinburgh Postnatal Depression Score | Felice, 2005[17] | Doubtful | Correlation between Maltese and English versions (r=0.95; p<0.001) | ? | ? | Very Low (High risk of bias due to doubtful methodological quality; Excellent response rate, low withdrawal rate, missing data not reported) |
| General Health Questionnaire-12 | | Not assessed | Not assessed | ? | ? | NA |
| Hopkins Symptom Checklist-10 | | Not assessed | Not assessed | ? | ? | NA |
| Hospital Anxiety and Depression Scale | | Not assessed | Not assessed | ? | ? | NA |
| Patient Health Questionnnaire-9 | | Not assessed | Not assessed | ? | ? | NA |
| Postpartum Depression Screening Scale | | Not assessed | Not assessed | ? | ? | NA |
| Zung Depression scale | | Not assessed | Not assessed | ? | ? | NA |
| **Reliability** | | | | | | |
| Beck Depression Inventory I | | Not assessed | Not assessed | ? | ? | NA |
| Beck Depression Inventory II | | Not assessed | Not assessed | ? | ? | NA |
| Center for Epidemiologic Studies Depression Scale | | Not assessed | Not assessed | ? | ? | NA |
| Edinburgh Postnatal Depression Score | Khalifa,[a] 2018[19] | Doubtful | Correlation coefficient=0.57; p< 0.001). Reliable change index of >1.96 | - | +/- | Low Mixed bias assessment, response rate not reported[3,12,27] |
| | Lau,[a] 2010[3] | Adequate | ICC=0.90 | + | | |
| | Montazeri, 2007[12] | Very good | ICC=0.80 | + | | |
| | Rowel, 2008[27] | Very good | ICC=0.96 (95% CI=0.86 - 0.99). | + | | |
| | Thompson, 1998[28] | Doubtful | Unclear of stability, Pearson correlation of 0.75-0.76 | ? | | |
| General Health Questionnaire-12 | Aguado, 2012[5] | Adequate | ICC 0.52-0.89 | ? | ? | Low Serious risk of bias due to methodology assessment, response rate not reported |
| Hopkins Symptom Checklist-10 | Khalifa,[a] 2018[19] | Doubtful | Reliable change index of ≥1.85 by HSCL-10 | ? | ? | Very low Very low risk of bias. Good sample size and response rate known, no missing data reported. |
| Hospital Anxiety and Depression Scale | | Not assessed | Not assessed | ? | ? | NA |
| Patient Health Questionnnaire-9 | | Not assessed | Not assessed | ? | ? | NA |

© 2022 Sultan P et al. *JAMA Network Open*.

| | | | | | | |
|---|---|---|---|---|---|---|
| Postpartum Depression Screening Scale | Pereira,[a] 2013[26] | Very good | ICC >0.7 from loss of appetite | + | + | Moderate (Convenience sample, good sample size good response rate, no missing data reported) |
| Zung Depression scale | Condon,[a] 1997[29] | Not assessed | Not assessed | ? | ? | NA |
| **Measurement error** | | | | | | |
| Beck Depression Inventory I | | Not assessed | Not assessed | ? | ? | NA |
| Beck Depression Inventory II | | Not assessed | Not assessed | ? | ? | NA |
| Center for Epidemiologic Studies Depression Scale | | Not assessed | Not assessed | ? | ? | NA |
| Edinburgh Postnatal Depression Score | | Not assessed | Not assessed | ? | ? | NA |
| General Health Questionnaire-12 | | Not assessed | Not assessed | ? | ? | NA |
| Hopkins Symptom Checklist-10 | | Not assessed | Not assessed | ? | ? | NA |
| Hospital Anxiety and Depression Scale | | Not assessed | Not assessed | ? | ? | NA |
| Patient Health Questionnnaire-9 | | Not assessed | Not assessed | ? | ? | NA |
| Postpartum Depression Screening Scale | | Not assessed | Not assessed | ? | ? | NA |
| Zung Depression scale | | Not assessed | Not assessed | ? | ? | NA |
| **Criterion Validity** | | | | | | |
| Beck Depression Inventory I | Harris,[a] 1989[30] | Very good | 68% Sensitivity; 88% specificity | ? | + | Moderate (Convenience sample, good response rate, missing data not reported) |
| | Ji,[a] 2011[31] | Very good | AUCs, ranging from 0.855 to 0.971, were all statistically significant (p<0.0001). | + | | |
| Beck Depression Inventory II | Beck,[a] 2001[4] | Very good | Major depression AUC 0.98 | + | + | Moderate (Convenience sampling, response rate not reported, [1] [4] good sample size, missing data not reported) |
| | Chaudron,[a] 2010[32] | Very good | AUCs for the BDI, EPDS, and PDSS for MDD were 0.84 (95% CI 0.78– 0.89), 0.86 (95% CI 0.81– 0.91), and 0.83 (95% CI 0.79 – 0.89), respectively, | + | | |
| | Wan Mahmud,[a] 2004[1] | Very good | AUC=0.995. | + | | |
| Center for Epidemiologic Studies Depression Scale | Beeghly, 2002[33] | Very good | Average *r*=0.52 (intake–3 month *r*=0.61, intake–6 months *r*= 0.46, intake–12 months *r*=0.38, 3–6 months *r*=0.65, 3–12 months *r*= 0.48, 6–12 months *r*=0.46, all *P* values ,0.0001) | - | +/- | Moderate (Good sample size Eligibility criteria not presented[33], numbers lost to follow up not reported[34]) |
| | Sasaki,[a] 2019[34] | Very good | ROC curve for each screening tool. (a) 4D-EPDS: AUC=0.800. (b) 1M-EPDS: AUC=0.797. (c) 1M-CES-D: AUC=0.765 | + | | |
| Edinburgh Postnatal Depression Score | Alvarado-Esquivel, 2006[35] | Very good | AUC=0.80 (DSM-IV interview) | + | + | Moderate (Good sample size in majority of studies (16 studies), good response rate in 1 study[29] not stated in 1 study,[1] poor response rate in 2 studies[21,32] |
| | Bunevicius, 2009[13] | Very good | 0.83 AUC (CIDI-SF) | + | | |
| | Chaudron,[a] 2010[32] | Very good | AUCs for the BDI, EPDS, and PDSS for MDD were 0.84 (95% CI 0.78– 0.89), 0.86 (95% CI 0.81– 0.91), and 0.83 (95% CI 0.79 – 0.89), respectively | + | | |

© 2022 Sultan P et al. *JAMA Network Open*.

| | | | | | | |
|---|---|---|---|---|---|---|
| | Chibanda, 2010[14] | Very good | AUC=0.82 | + | | |
| | Cox, 1987[9] | Very good | RDC- sensitivity 85% and specificity 77% | ? | | |
| | Felice, 2005[17] | Very good | AUC=0.95 | + | | |
| | Garcia-Esteve, 2003[36] | Very good | AUC= 0.976 (DSM-III) | + | | |
| | Hanusa,[a] 2008[37] | Very good | ROC EPDS, PHQ-9 and PDSS 0.84, 0.79, 0.73 | + | | |
| | Harris,[a] 1989[30] | Very good | 95% sensitivity- 93% Specificity | ? | | |
| | Ji,[a] 2011[31] | Very good | AUCs, ranging from 0.855 to 0.971, were all statistically significant (p<0.0001). | + | | |
| | Lau,[a] 2010[3] | Very good | AUC 89.6% for EPDS vs clinical depression | + | | |
| | Lee,[a] 2000[38] | Very good | Sens 0.88 spec 0.89 cut off of 4.5 for GHQ. Sens 0.82 spec 0.86 cut off of 9.5 for EDPS | ? | | |
| | Mazhari,[a] 2007[20] | Very good | Major depression (cut-off 12/13) with a sensitivity and specificity of 95.3% and 87.9% | ? | | |
| | Murray,1990 [39] | Very good | Major depression EPDS score of 10.5 88.3% specificity, 92.6% | ? | | |
| | Navarro,[a] 2007[40] | Very good | AUC=0.9 (SCID) | + | | |
| | Pitanupong, 2007[21] | Very good | AUC 0.84 (95% confidence interval 0.76–0.91). | + | | |
| | Rowel, 2008[27] | Very good | ICD-10 clinical examination. ROC 0.94 (95% CI=0.91-0.98; SE=0.02). | + | | |
| | Sasaki,[a] 2019[34] | Very good | The ROC curve for each screening tool. (a) 4D-EPDS: AUC = 0.800. (b) 1M-EPDS: AUC=0.797. (c) 1M-CES-D: AUC=0.765. | + | | |
| | Smith-Nielsen, 2019[4] | Very good | EPDS vs ICD AUC= 0.960 | + | | |
| | Teng, 2005[22] | Very good | AUC=0.97 | + | | |
| | Thompson,[a] 1998[28] | Very good | EPDS 84 spec 95 sensitivity for definite RDC | ? | | |
| | Wan Mahmud,[a] 2004[1] | Very good | AUC=0.995. | + | | |
| | Yonkers, 2001 [41] | Very good | Sensitivity 78% specificity 90% of major depression with a threshold of 11 | ? | | |
| General Health Questionnaire-12 | Lee,[a] 2000[38] | Very good | Sens 0.88 spec 0.89 cut off of 4.5 for GHQ. Sens 0.82 spec 0.86 cut off of 9.5 for EDPS | ? | + | Moderate (Good sample size, loss to follow up[38] and missing data not reported) |
| | Navarro,[a] 2007[40] | Very good | AUC=0.904 (SCID) | + | | |
| Hopkins Symptom Checklist-10 | Khalifa[a] 2018[19] | Not assessed | Not assessed | ? | ? | NA |
| Hospital Anxiety and Depression Scale | Thompson,[a] 1998[28] | Very good | 90% spec 65% sensitivity for major depression | ? | ? | Low (Convenience sample, loss to follow up and missing data not reported) |
| Patient Health Questionnnaire-9 | Hanusa,[a] 2008[37] | Very good | ROC EPDS, PHQ-9 and PDSS 0.84, 0.79, 0.73 | + | + | Low (Convenience sample and low sample size, good response rate missing data not reported) |
| Postpartum Depression Screening Scale | Beck,[a] 2001[4] | Very good | Major depression AUC 0.98 | + | + | Moderate (Number of eligible patients not known[6] poor response rate[23] missing data not reported in all studies) |
| | Lara,[a] 2013[23] | Very good | AUC for major depression 0.88 SD 0.05 | + | | |
| | Pereira,[a] 2013[26] | Very good | AUC 0.98 for mild. 0.93 for major depression | + | | |
| Zung Depression scale | | Not assessed | Not assessed | ? | ? | NA |

© 2022 Sultan P et al. *JAMA Network Open*.

| | | | | | | |
|---|---|---|---|---|---|---|
| **Hypothesis testing (comparison of scores between measures / group comparisons)** | | | | | | |
| Beck Depression Inventory I | Harris,[a] 1989[30] | Adequate | Intercorrelation 0.68 BDI and EPDS | + | + | Moderate<br>(Low risk of bias, good sample size, missing data not reported in any study, good response rate, unknown numbers eligible[3]) |
| | Lau,[a] 2010[3] | Very good | Correlation between BDI and EPDS 0.349. Mean SD) 115.4 (15.86) | + | | |
| Beck Depression Inventory II | Beck,[a] 2001[4] | Very good | PDSS Correlated with EPDS and BDI R 0.79 and 0.81 with SD & 4.93 and 7.25 respectively | + | + | Moderate<br>(Low risk of bias, good sample sizes, missing data not reported in any study, low response rate [23], unknown eligibility[1]) |
| | Lara,[a] 2013[23] | Very good | Correlation was adequate, compared with the BDI-II (T1: *r* = 0.75; T2: *r* = 0.74, and the SCID (T1: *r* = 0.43; T2: *r* = 0.36,) SD = 0.02; | + | | |
| | Pereira,[a] 2013[26] | Very good | PDSS and BDI-II total score was high and significant (r=0.63) SD | + | | |
| | Wan Mahmud,[a] 2004[1] | Very good | Correlation between BDI and EPDS 0.74 SD mean for both reported | + | | |
| Center for Epidemiologic Studies Depression Scale | Sasaki,[a] 2019[34] | Very good | No significant difference was found in the sensitivity, positive predictive value or negative predictive value among the three screening methods. (Mean and SD reported) | + | + | Low<br>(Low risk of bias, low sample size) |
| Edinburgh Postnatal Depression Score | Bassi, 2017[8] | Adequate | Depression was significantly and negatively correlated with the global psychological well-being scores (r=–0.38; Mean/ SD reported) | + | + | Moderate<br>(Low risk of bias, good sample size[29], response rate not reported,[1]<br>Poor response rate[21,32]) |
| | Beck,[a] 2001[4] | Very good | PDSS Correlated with EPDS and BDI r= 0.79 and 0.81 | + | | |
| | Condon,[a] 1997[29] | Adequate | Correlation between EPDS, HADs, POMs-D and Zung at 3 time points between 0.57 and 0.73 mean SD scores | + | | |
| | Hanusa,[a] 2008[37] | Very good | Correlations between the three measures were EPDS with PHQ-9 (*r*= 0.75), EPDS with PDSS_SF (*r* =0.75,), and PHQ-9 with PDSS_SF (*r*= 0.71) dichotomised data | + | | |
| | Harris,[a] 1989[30] | Adequate | Correlation r=0.68 BDI and EPDS no distribution data | + | | |
| | Khalifa,[a] 2018[19] | Very good | 3-month correlation between ESPD and total HSCL-10 score 0.77 (+) and 8-month 0.78 (+) means SD scores over time | + | | |
| | Lau,[a] 2010[3] | Very good | Correlation between BDI and EPDS 0.349 mean and SD | + | | |
| | Lee,[a] 2000[38] | Adequate | Correlation coefficient with GHQ-12 total score was 0.76 no mean | + | | |
| | Mazhari,[a] 2007[20] | Very good | The correlation coefficient of the total score of the Persian version of EPDS with the GHQ-12 total score was 0.76 mean (S.D) 17.83 (3.75), | + | | |
| | Mwape, 2019[42] | Adequate | Non- significant correlation between EDPS and GHQ. Means but no SD | + | | |
| | Navarro,[a] 2007[40] | Very good | The EPDS showed a high correlation with the GHQ-12 ($r^2$=0.80; Pb.0001). SD recorded | + | | |

© 2022 Sultan P et al. *JAMA Network Open*.

| | | | | | | |
|---|---|---|---|---|---|---|
| | Sasaki,[a] 2019[34] | Very good | No significant difference was found in the sensitivity, positive predictive value or negative predictive value among the three screening methods. Mean and SD reported | + | | |
| | Thompson, 1998[28] | Adequate | EPDS and Hamilton rating scale for depression at 8/12/20/28 weeks 0.76/0.78/0.78/0.75 no distribution data | + | | |
| | Wan Mahmud,[a] 2004[1] | Very good | Corelation between BDI and EPDS 0.74 SD mean for both reported | + | | |
| General Health Questionnaire-12 | Mazhari,[a] 2007[20] | Very good | Correlation coefficient of the total score of the Persian version of EPDS with the GHQ-12 total score was 0.76 mean (SD) 17.83 (3.75) | + | + | Moderate (Low risk of bias, good sample size response rate not reported[42] no missing data analysis) |
| | Mwape, 2019[42] | Adequate | Non-significant correlation between EDPS and GHQ Means but no SD presented | + | | |
| | Navarro,[a] 2007[40] | Very good | The EPDS showed a high correlation with the GHQ-12 $r^2$=0.80; SD recorded | + | | |
| Hopkins Symptom Checklist-10 | Khalifa,[a] 2018[19] | Very good | 3month correlation between ESPD and total HSCL-10 score0.77 and 8 month 0.78 means SD scores over time. | + | + | Moderate (Low risk of bias, good sample size, good response rate, no missing data reported) |
| Hospital Anxiety and Depression Scale | Condon,[a] 1997[29] | Very good | Correlation between EPDS, HADs, POMs-D and Zung at 3 time points between 0.57 and 0.73 mean SD scores | + | + | Low (Low risk of bias, good sample size, no response rate reported,[29] no missing data reported) |
| | Thompson, 1998[28] | Adequate | r-value EPDS and Hamilton rating scale for depression at 8/12/20/28 weeks 0.76/0.78/0.78/0.75 no distribution data | + | | |
| Patient Health Questionnnaire-9 | Hanusa,[a] 2008[37] | Adequate | Correlations between the three measures were EPDS with PHQ-9 (*r*= 0.75), EPDS with PDSS_SF (*r* =0.75), and PHQ-9 with PDSS_SF (*r*= 0.71) Dichotomised data | + | + | Low (Serious risk of bias, small sample size, low response rate, no missing data reported) |
| Postpartum Depression Screening Scale | Beck,[a] 2001[4] | Very good | PDSS Correlated with EPDS and BDI R 0.79 and 0.81 | + | + | Moderate (Low risk of bias, good sample size, low response rate[23], response rate not known,[4] no missing data reported) |
| | Lara,[a] 2013[23] | Very good | Correlation with the BDI-II (T1: *r* = 0.75; T2: *r* = 0.74, *ps* < 0.01) and the SCID (T1: *r* = 0.43; T2: *r* = 0.36, SD = 0.02; | + | | |
| | Pereira,[a] 2013[26] | Very good | PDSS and BDI-II total score was high and significant ($r_S$=0.63) SD | + | | |
| Zung Depression scale | Condon,[a] 1997[29] | Very good | Correlation between EPDS, HADs, POMs-D and Zung at 3 time points between 0.57 and 0.73 mean SD scores | + | + | Low (Low risk of bias, response rate not reported) |
| **Responsiveness** | | | | | | |
| Beck Depression Inventory I | | Not assessed | Not assessed | ? | ? | NA |
| Beck Depression Inventory II | Lara,[a] 2013[23] | Very good | Pearson's correlations PDSS and BDI-II were *r* = 0.75(T1), and *r*=0.74 (T2) SD | + | + | Low (Low risk of bias, good sample size, poor response rate. Follow up and missing data not reported) |

© 2022 Sultan P et al. *JAMA Network Open*.

| | | | | | | |
|---|---|---|---|---|---|---|
| Center for Epidemiologic Studies Depression Scale | Beeghly, 2002[33] | Very good | Average *r*=0.52; intake–3-month *r*=0.61, intake–6 months *r*=0.46, intake–12 months *r*=0.38, 3–6 months *r*=0.65, 3–12 months *r*=0.48, 6–12 months *r*=0.46, Mean and SD scores | ? | ? | Moderate (Low risk of bias, good sample size, and response rate, missing data not reported) |
| Edinburgh Postnatal Depression Score | Condon,[a] 1997[29] | Very good | Range, mean and SD between 4 weeks and 8 months for EPDS, HADS-D and Zung | + | + | Moderate (Low risk of bias, good sample size, variable response rates, loss to follow up not reported in all studies, missing data reported in 1 study[11]) |
| | Coo, 2015[10] | Very good | No change in EPDS scores between 3 time points (D7-10, and 10-12 weeks) 6.99 (4.11) to 6.19 (4.58) | + | | |
| | Doering Runquist, 2009[11] | Very good | Fatigue and depressive symptoms were moderately to strongly correlated at 1 (r = 0.68), 3 (r = 0.74), and 6 (r = 0.70) months postpartum | + | | |
| | Glavin, 2010[18] | Very good | Significant differences in mean EPDS score between groups after the intervention at 3 months (P<0.001), and again at 6 months (P<0.001) | + | | |
| | Heron, 2010[43] | Very good | 8-week EPDS scores were significantly lower than postnatal scores ( z=4.462,p < 0.0001),at one week postnatal (mean score=7.84), at 8 weeks (mean score=6.41) + | + | | |
| | Khalifa,[a] 2018[19] | Very good | 3-month correlation between EPDS and total HSCL-10 score 0.77 (+) and 8 month 0.78 (+). EPDS and HSCL difference in means 1.83 and 0.13 respectively P <0.001 | + | | |
| | Sasaki,[a] 2019[34] | Very good | Day 4 and 1month EPDS sensitivity and specificity scores and diagnostic rate | + | | |
| | Thompson,[a] 1998[28] | Very good | r-value EPDS and Hamilton rating scale for depression at 8/12/20/28 weeks 0.76/0.78/0.78/0.75 | + | | |
| General Health Questionnaire-12 | | Not assessed | Not assessed | ? | ? | NA |
| Hopkins Symptom Checklist-10 | Khalifa,[a] 2018[19] | Very good | 3-month correlation between EPDS and total HSCL-10 score 0.77 (+) and 8 month 0.78 (+). EPDS and HSCL difference in means 1.83 and 0.13 respectively P <0.001 | + | + | Moderate (Low risk of bias, good response rate, missing data not known) |
| Hospital Anxiety and Depression Scale | Condon,[a] 1997[29] | Very good | Range, mean and SD between 4 weeks and 8 months for EPDS, HADS-D and Zung | + | + | Moderate (Low risk of bias, good response rate, missing data not reported) |
| Patient Health Questionnnaire-9 | | Not assessed | Not assessed | ? | ? | NA |
| Postpartum Depression Screening Scale | Lara,[a] 2013[23] | Very good | PDSS were significantly correlated at T1 (r = 0.89, p <0 .01) and T2 (r = 0.87, p <0 .01). | + | + | Low (Low risk of bias, however low response rate, high loss to follow up and missing data not reported) |
| Zung Depression scale | Condon,[a] 1997[29] | Very good | Range, mean and SD between 4 weeks and 8 months for EPDS, HADS-D and Zung | + | + | Moderate (Low risk of bias, good response rate, missing data not reported) |

a=denotes a study which psychometrically evaluates >1 PROM; Methods rating: Very good, Adequate, Doubtful, Inadequate or Not assessed (NA); Overall rating: Sufficient (+), Insufficient (-), Inconsistent (+/-) or Indeterminate (?); SD=standard deviation;

© 2022 Sultan P et al. *JAMA Network Open*.

ICC=interclass correlation coefficient; AUC=area under the curve; CFA=confirmatory factor analysis; CFI=comparative fit index; RMSEA=root mean square error of approximation, SRMR=standardized root mean residuals; TLI=Tucker-Lewis Index; SCID=structured clinical interview for DSM-5; BDI=Beck Depression Inventory; CESDS=Center for Epidemiologic Studies Depression Scale; EPDS= Edinburgh Postnatal Depression Score; GHQ-12= General Health Questionnaire-12; HSC10=Hopkins Symptom Checklist-10; HADS-D= Hospital Anxiety and Depression Scale; PHQ-9=Patient Health Questionnnaire-9; PDSS=Postpartum Depression Screening Scale; Zung= Zung Depression scale; T1 =time point 1; D=day; NRS=numerical rating score; VAS=visual analog score; Sample size described as per COSMIN criteria (good >100; low 50-100 (-1 GRADE rating); very low <50 (-2 GRADE rating) associated with imprecision as described by modified GRADE approach); survey response rate ≥60% was considered good.

© 2022 Sultan P et al. *JAMA Network Open*.

## eReferences

1. Wan Mahmud WMR, Awang A, Herman I, Mohamed MN. Analysis of the psychometric properties of the Malay version of Beck Depression Inventory II (BDI-II) among postpartum women in Kedah, North West of Peninsular Malaysia. *Malaysian J Med Sci*. 2004;11(2):19-25.
2. Giallo R, Gartl, Woolhouse H, Brown S. Differentiating maternal fatigue and depressive symptoms at six months and four years post partum: Considerations for assessment, diagnosis and intervention. *Midwifery*. 31(2):316-322.
3. Lau Y, Wang Y, Yin L, Chan KS, Guo X. Validation of the Mainland Chinese version of the Edinburgh Postnatal Depression Scale in Chengdu mothers. *Int J Nurs Stud*. 2010;47(9):1139-1151. doi:10.1016/j.ijnurstu.2010.02.005
4. Smith-Nielsen J, Matthey S, Lange T, Væver MS. Validation of the Edinburgh Postnatal Depression Scale against both DSM-5 and ICD-10 diagnostic criteria for depression. *BMC Psychiatry*. 2018;18(1):1-12. doi:10.1186/s12888-018-1965-7
5. Aguado J, Campbell A, Ascaso C, Navarro P, Garcia-Esteve L, Luciano J V. Examining the Factor Structure and Discriminant Validity of the 12-Item General Health Questionnaire (GHQ-12) Among Spanish Postpartum Women. *Assessment*. 2012;19(4):517-525. doi:10.1177/1073191110388146
6. Beck CT, Gable RK. Postpartum depression screening scale: Spanish version. *Nurs Res*. 2003;52(5):296-306. doi:10.1097/00006199-200309000-00004
7. Blucker RT, Gillaspy JA, Jackson D, et al. Postpartum depression in the NICU: An examination of the factor structure of the postpartum depression screening scale. *Adv Neonatal Care*. 2014;14(6):424-432. doi:10.1097/ANC.0000000000000135
8. Bassi M, Delle Fave A, Cetin I, et al. Psychological well-being and depression from pregnancy to postpartum among primiparous and multiparous women. *J Reprod Infant Psychol*. 2017;35(2):183-195.
9. Cox J, Holden J, Sagovsky R. Detection of Postnatal Depression Development of the 10-item Edinburgh Postnatal Depression Scale. *Br J Psychiatry*. 1987;150:782-786.
10. Coo S, Milgrom J, Kuppens P, Trinder J. Perinatal distress, an appraisal perspective. *J Reprod Infant Psychol*. 2015;33(2):190-204.
11. Doering JJ, Morin K, Stetzer FC. Severe fatigue and depressive symptoms in lower-income urban postpartum women. *West J Nurs Res*. 2009;31(5):599-612. doi:10.1177/0193945909333890
12. Montazeri A, Torkan B, Omidvari S. The Edinburgh Postnatal Depression Scale (EPDS): translation and validation study of the Iranian version. *BMC Psychiatry*. 2007;7:11. doi:10.1186/1471-244X-7-11
13. Bunevičius A, Kusminskas L, Bunevičius R. Validation of the Lithuanian version of the Edinburgh postnatal depression scale. *Medicina (B Aires)*. 2009;45(7):544-548. doi:10.3390/medicina45070072
14. Chibanda D, Mangezi W, Tshimanga M, et al. Validation of the Edinburgh Postnatal Depression Scale among women in a high HIV prevalence area in urban Zimbabwe. *Arch Womens Ment Health*. 2010;13(3):201-206. doi:10.1007/s00737-009-0073-6
15. Dørheim S, Bjorvatn B, Eberhard-Gran M. Can Insomnia in Pregnancy Predict Postpartum Depression? A Longitudinal, Population-Based Study. *PLoS One*. 2014;9(4):e94674.
16. Edhborg M, Nasreen H-E, Kabir Z. Impact of postpartum depressive and anxiety

© 2022 Sultan P et al. *JAMA Network Open*.

symptoms on mothers' emotional tie to their infants 2-3 months postpartum: a population-based study from rural Bangladesh. *Arch Womens Ment Health*. 2011;14(4):307-316.
17. Felice E, Saliba J, Grech V, Cox J. Validation of the Maltese version of the Edinburgh Postnatal Depression Scale. *Arch Womens Ment Health*. 2006;9(2):75-80. doi:10.1007/s00737-005-0099-3
18. Glavin K, Smith L, Sorum R, Ellefsen B. Supportive counselling by public health nurses for women with postpartum depression. *J Adv Nurs*. 66(6):1317-1327.
19. Khalifa DS, Glavin K, Bjertness E, Lien L. Course of depression symptoms between 3 and 8months after delivery using two screening tools (EPDS and HSCL-10) on a sample of Sudanese women in Khartoum state. *BMC Pregnancy Childbirth*. 2018;18(1). doi:10.1186/s12884-018-1948-1
20. Mazhari S, Nakhaee N. Validation of the Edinburgh Postnatal Depression Scale in an Iranian sample. *Arch Womens Ment Health*. 2007;10(6):293-297. doi:10.1007/s00737-007-0204-x
21. Pitanupong J, Liabsuetrakul T, Vittayanont A. Validation of the Thai Edinburgh Postnatal Depression Scale for screening postpartum depression. *Psychiatry Res*. 2007;149(1-3):253-259. doi:10.1016/j.psychres.2005.12.011
22. Teng HW, Hsu C Sen, Shih SM, Lu ML, Pan JJ, Shen WW. Screening postpartum depression with the Taiwanese version of the Edinburgh Postnatal Depression Scale. *Compr Psychiatry*. 2005;46(4):261-265. doi:10.1016/j.comppsych.2004.10.003
23. Lara MA, Navarrete L, Navarro C, Le H-N. Evaluation of the Psychometric Measures for the Postpartum Depression Screening Scale–Spanish Version for Mexican Women. *J Transcult Nurs*. 2013;24(4):378-386. doi:10.1177/1043659613493436
24. Beck CT, Gable RK. Postpartum depression screening scale: Development and psychometric testing. *Nurs Res*. 2000;49(5). doi:10.1097/00006199-200009000-00006
25. Beck CT, Gable RK. Further validation of the postpartum depression screening scale. *Nurs Res*. 2001;50(3):155-164. doi:10.1097/00006199-200105000-00005
26. Pereira AT, Bos S, Marques M, et al. The Portuguese version of the postpartum depression screening scale. *J Psychosom Obstet Gynecol*. 2010;31(2):90-100. doi:10.3109/0167482X.2010.481736
27. Rowel D, Jayawardena P, Fernando N. Validation of the Sinhala translation of Edinburgh Postnatal Depression Scale. *Ceylon Med J*. 2008;53(1):10-13. doi:10.4038/cmj.v53i1.219
28. Thompson WM, Harris B, Lazarus J, Richards C. A comparison of the performance of rating scales used in the diagnosis of postnatal depression. *Acta Psychiatr Scand*. 1998;98(3):224-227. doi:10.1111/j.1600-0447.1998.tb10071.x
29. Condon JT, Corkindale CJ. The assessment of depression in the postnatal period: A comparison of four self-report questionnaires. *Aust N Z J Psychiatry*. 1997;31(3). doi:10.3109/00048679709073844
30. Harris B, Huckle P, Thomas R, Johns S, Fung H. The use of rating scales to identify post-natal depression. *Br J Psychiatry*. 1989;154(JUN.). doi:10.1192/bjp.154.6.813
31. Ji S, Long Q, Newport DJ, et al. Validity of depression rating scales during pregnancy and the postpartum period: impact of trimester and parity. *J Psychiatr Res*. 2011;45(2):213-219. doi:10.1016/j.jpsychires.2010.05.017
32. Chaudron LH, Szilagyi PG, Tang W, et al. Accuracy of depression screening tools for identifying postpartum depression among urban mothers. *Pediatrics*. 2010;125(3):e609-17. doi:10.1542/peds.2008-3261

© 2022 Sultan P et al. *JAMA Network Open*.

33. Beeghly M, Weinberg MK, Olson KL, Kernan H, Riley J, Tronick EZ. Stability and change in level of maternal depressive symptomatology during the first postpartum year. *J Affect Disord*. 2002;71(1-3):169-180. doi:10.1016/S0165-0327(01)00409-8
34. Sasaki Y, Baba T, Oyama R, Fukumoto K, Haba G, Sasaki M. Re-evaluation of the Edinburgh Postnatal Depression Scale as screening for post-partum depression in Iwate Prefecture, Japan. *J Obstet Gynaecol Res*. 2019;45(9):1876-1883. doi:10.1111/jog.14042
35. Alvarado-Esquivel C, Sifuentes-Alvarez A, Salas-Martinez C, Martínez-García S. Validation of the Edinburgh postpartum depression scale in a population of puerperal women in Mexico. *Clin Pract Epidemiol Ment Heal*. 2006;2. doi:10.1186/1745-0179-2-33
36. Garcia-Esteve L, Ascaso C, Ojuel J, Navarro P. Validation of the Edinburgh Postnatal Depression Scale (EPDS) in Spanish mothers. *J Affect Disord*. 2003;75(1):71-76. doi:10.1016/S0165-0327(02)00020-4
37. Hanusa BH, Scholle SH, Haskett RF, Spadaro K, Wisner KL. Screening for depression in the postpartum period: a comparison of three instruments. *J Womens Health (Larchmt)*. 2008;17(4):585-596. doi:10.1089/jwh.2006.0248
38. Lee DTS, Yip ASK, Chiu HFK, Chung TKH. Screening for postnatal depression using the double-test strategy. *Psychosom Med*. 2000;62(2):258-263. doi:10.1097/00006842-200003000-00018
39. Murray L, Carothers AD. The validation of the Edinburgh Post-natal Depression Scale on a community sample. *Br J Psychiatry*. 1990;157(3):288-290. doi:10.1192/bjp.157.2.288
40. Navarro P, García-Esteve L, Ascaso C, Aguado J, Gelabert E, Martín-Santos R. Non-psychotic psychiatric disorders after childbirth: Prevalence and comorbidity in a community sample. *J Affect Disord*. 2008;109(1):171-176.
41. Yonkers KA, Ramin SM, Rush AJ, et al. Onset and persistence of postpartum depression in an inner-city maternal health clinic system. *Am J Psychiatry*. 2001;158(11):1856-1863. doi:10.1176/appi.ajp.158.11.1856
42. Mwape L, Lyambai K, Chitundu K. Predicting Postnatal Psychological Distress in a Zambian Cohort: Relationship between the EPDS and the GHQ-12. *Open J Psychiatry*. 2019;09(01):39-52. doi:10.4236/ojpsych.2019.91004
43. Heron J, Haque S, Oyebode F, Craddock N, Jones I. A longitudinal study of hypomania and depression symptoms in pregnancy and the postpartum period. *Bipolar Disord*. 11(4):410-417.

© 2022 Sultan P et al. *JAMA Network Open*.
